# Characterization of the molecular changes associated with the overexpression of a novel epithelial cadherin splice variant mRNA in a breast cancer model using proteomics and bioinformatics approaches: identification of changes in cell metabolism and an increased expression of lactate dehydrogenase B

**DOI:** 10.1186/s40170-019-0196-9

**Published:** 2019-05-09

**Authors:** Marina Rosso, Lara Lapyckyj, María José Besso, Marta Monge, Jaume Reventós, Francesc Canals, Jorge Oswaldo Quevedo Cuenca, María Laura Matos, Mónica Hebe Vazquez-Levin

**Affiliations:** 1 Laboratorio de Estudios de Interacción Celular en Reproducción y Cáncer, Instituto de Biología y Medicina Experimental (IBYME; CONICET-FIBYME), Vuelta de Obligado 2490, C1428ADN Buenos Aires, Argentina; 2 Vall d’Hebron Institute of Oncology (VHIO), Barcelona, Spain; 3 Departament de Ciències Bàsiques, Universitat Internacional de Catalunya, Barcelona, Spain

**Keywords:** Breast cancer, Epithelial cadherin, Alternative splicing, Epithelial to mesenchymal transition, Proteomic analysis, 2D-DIGE, Mass spectrometry, Glycolysis, Lactate dehydrogenase B

## Abstract

**Background:**

Breast cancer (BC) is the most common female cancer and the leading cause of cancer death in women worldwide. Alterations in epithelial cadherin (E-cadherin) expression and functions are associated to BC, but the underlying molecular mechanisms have not been fully elucidated. We have previously reported a novel human E-cadherin splice variant (E-cadherin variant) mRNA. Stable transfectants in MCF-7 human BC cells (MCF7Ecadvar) depicted fibroblast-like cell morphology, E-cadherin wild-type downregulation, and other molecular changes characteristic of the epithelial-to-mesenchymal transition process, reduced cell-cell adhesion, and increased cell migration and invasion. In this study, a two-dimensional differential gel electrophoresis (2D-DIGE) combined with mass spectrometry (MS) protein identification and bioinformatics analyses were done to characterize biological processes and canonical pathways affected by E-cadherin variant expression.

**Results:**

By 2D-DIGE and MS analysis, 50 proteins were found differentially expressed (≥ Δ1.5) in MCF7Ecadvar compared to control cells. Validation of transcript expression was done in the ten most overexpressed and underexpressed proteins. Bioinformatics analyses revealed that 39 of the 50 proteins identified had been previously associated to BC. Moreover, metabolic processes were the most affected, and glycolysis the canonical pathway most altered. The lactate dehydrogenase B (LDHB) was the highest overexpressed protein, and transcript levels were higher in MCF7Ecadvar than in control cells. In agreement with these findings, MCF7Ecadvar conditioned media had lower glucose and higher lactate levels than control cells. MCF7Ecadvar cell treatment with 5 mM of the glycolytic inhibitor 2-deoxy-glucose led to decreased cell viability, and modulation of LDHB expression in MCF7Ecadvar cells with a specific small interfering RNA resulted in decreased cell proliferation. Finally, a positive association between expression levels of the E-cadherin variant and LDHB transcripts was demonstrated in 21 human breast tumor tissues, and breast tumor samples with higher Ki67 expression showed higher LDHB mRNA levels.

**Conclusions:**

Results from this investigation contributed to further characterize molecular changes associated to the novel E-cadherin splice variant expression in BC cells. They also revealed an association between expression of the novel variant and changes related to BC progression and aggressiveness, in particular those associated to cell metabolism.

**Electronic supplementary material:**

The online version of this article (10.1186/s40170-019-0196-9) contains supplementary material, which is available to authorized users.

## Background

Breast cancer (BC) is the most common cancer in women, representing 25% of female cancers. Moreover, it is the fifth cause of cancer death and women death leading cause by cancer [1]. Taking into account the high incidence and mortality associated to BC, it is still a great challenge to delve into the molecular mechanisms involved in the origin and progression of this heterogeneous disease, to better understand its pathogenesis and to identify novel biomarkers and new therapeutic targets.

Epithelial cadherin (E-cadherin) is a calcium-ion dependent cell-cell adhesion molecule that plays a key role in the maintenance of normal epithelia intercellular junctions and in a variety of processes fundamental to tissue homeostasis, such as differentiation, proliferation, migration, cell death, and survival [2]. A partial or total loss of E-cadherin expression and/or function(s) has been reported in numerous tumors of epithelial origin, among them in breast tumors, leading to destabilization of cell-cell interactions and aberrant activation of cellular signaling pathways [3, 4]. A reduced expression of E-cadherin is a hallmark of epithelial-mesenchymal transition (EMT), a process that induces changes in gene expression and in cell behavior that have been associated with tumor progression and aggressiveness [5]. Despite its relevance, the underlying molecular alterations leading to deregulation of E-cadherin expression and function(s) have not been completely characterized. Among the most frequent mechanisms responsible of these changes are a loss of heterozygosity, mutations, epigenetic silencing, expression of transcriptional repressors, and post-translational modifications [6, 7]. In recent years, a differential regulation of alternative splicing (AS) and its association with molecular and phenotypic changes has been reported during EMT [8, 9]. Under normal conditions, AS regulates gene expression and contributes to the expansion of proteomic diversity in a spatially and temporally controlled fashion. However, during malignant transformation, AS events have been reported in tumor suppressor genes or oncogenes, which result in association with mutations at specific splice sites and/or with changes in the expression of AS regulatory factors, among other causes, changes that contribute to tumor progression and the acquisition of therapeutic resistance [10, 11].

We have recently reported, for the first time, a novel transcript of human E-cadherin that differs from the wild-type E-cadherin (E-cadherin wt) mRNA by lacking the first 34 bp of Exon 14. This novel variant, called E-cadherin variant, would result from an AS event of the immature E-cadherin mRNA. Its expression levels would be regulated, at least in part, by the nonsense-mediated mRNA decay (NMD) surveillance mechanism. The E-cadherin variant mRNA was detected in cell lines derived from different human tumors types. Moreover, stable transfection of the E-cadherin variant in the MCF-7 human BC cell line (MCF7Ecadvar) induces a significant decrease in the E-cadherin wt (transcript and protein) expression, as well as changes that resemble the EMT process. MCF7Ecadvar cells depict a fibroblast-like morphology, and show changes in the expression levels of several EMT markers (i.e. cytokeratins, vimentin, and neural cadherin), reduced cell-cell interaction, increased cell migration and cell invasive properties when compared to the control cell line [12].

The present study aimed to thoroughly characterize molecular changes associated to the novel E-cadherin splice variant mRNA expression in MCF-7 cells, by performing a proteomic analysis using the two-dimensional differential gel electrophoresis (2D-DIGE) technology combined with protein identification by mass spectrometry (MS). In addition, this study aimed to identify other cellular processes and cell signaling pathways affected by the expression of the novel E-cadherin splice variant mRNA using bioinformatics tools, and to compare these changes with those previously associated with BC. Finally, studies on the lactate dehydrogenase B chain (LDHB) enzyme were done; this protein was identified in the proteomic analysis with the highest increased expression in MCF7Ecadvar compared to MCF7pcDNA3 control cells.

## Results

### 2D-DIGE and MS proteomic analysis of MCF7Ecadvar cells

To assess the global protein profile of MCF-7 cells stably transfected with the novel E-cadherin variant transcript, an expression analysis was carried out using the 2D-DIGE technique coupled to protein identification by MS. For this purpose, MCF7Ecadvar and MCF7pcDNA3 cell cultures (three per cell line) were prepared and processed by means of total protein precipitation and quantification, as detailed in “Materials and Methods” section. Downregulation of E-cadherin wt in MCF7Ecadvar cells was confirmed by Western immunoblotting of total protein extracts (Additional file 1: Figure S1).

Total protein extracts from MCF7pcDNA3 and MCF7Ecadvar cells were then labeled with fluorescent dyes, mixed, and separated by isoelectric focusing (first-dimension) followed by denaturing polyacrylamide gel electrophoresis (second-dimension). After gel scanning, protein alignment, and analysis, 64 protein spots were identified as differentially expressed between both cell lines (1.5-fold change cutoff and an analysis of variance (ANOVA) ≤ 0.005). Of these spots, 60 were recovered and their spectra were obtained by MS. The identity of each protein spot was determined using the Mascot Database Search Program (Matrix Science, London, UK). As result of these studies, 50 proteins were identified: ten proteins were found overexpressed and 40 underexpressed in MCF7Ecadvar cells relative to MCF7pcDNA3 cells. The name and symbol of these proteins, their UniProt number, the symbol of the gene encoding each protein, a brief protein functional description, and the fold change value associated to each protein are included in Table 1.

**Table 1:** Proteins identified as differentially expressed between MCF7Ecadvar and MCF7pcDNA3 cells

Protein name	Protein symbol	UniProt number	Gene symbol	Protein function	Fold
l-lactate dehydrogenase B chain	LDHB	P07195	LDHB	Catalysis of the interconversion of pyruvate and lactate with concomitant interconversion of NADH and NAD+ in a post-glycolysis process.	9.34
Aldose reductase	ALDR	P15121	AKR1B1	Catalysis of the NADPH-dependent reduction of a wide variety of carbonyl-containing compounds to their corresponding alcohols with a broad range of catalytic efficiencies.	4.49
Elongation factor 2	EF2	P13639	EEF2	Catalysis of the GTP-dependent ribosomal translocation step during translation elongation. Catalysis of the coordinated movement of the two tRNA molecules, the mRNA and conformational changes in the ribosome.	3.94
Heat shock protein HSP 90-beta	HS90B	P08238	HSP90AB1	Molecular chaperone. Promotion of the maturation, structural maintenance, and regulation of specific target proteins involved in cell cycle control and signal transduction. ATPase activity. Regulation of the transcription machinery at different levels: alteration of certain transcription factors levels in response to physiological cues, modulation of certain epigenetic modifiers, and participation in the eviction of histones from the promoter region of specific genes to turn on gene expression.	3.42
14-3-3 protein theta	1433 T	P27348	YWHAQ	Adapter protein. Regulation of a large spectrum of both general and specialized signaling pathways. Binding to a large number of partners, usually by recognition of a phosphoserine or phosphothreonine motif, which results in the modulation of the binding partner activity.	3.36
Vimentin	VIME	P08670	VIM	Class-III intermediate filament found in diverse non-epithelial cells, especially mesenchymal cells. Attached to the nucleus, endoplasmic reticulum and mitochondria, either laterally or terminally.	3.05
Ubiquitin-like modifier-activating enzyme 1	UBA1	P22314	UBA1	Catalysis of the first step in ubiquitin conjugation to mark cellular proteins for degradation through the ubiquitin-proteasome system. Essential for the formation of radiation-induced foci, timely DNA repair and response to replication stress.	2.66
Protein disulfide-isomerase A3	PDIA3	P30101	PDIA3	Prevention of the formation of protein aggregates. Catalysis of the rearrangement of -S-S- bonds in proteins. Complexes of lectins and this protein mediate protein folding by promoting formation of disulfide bonds in their glycoprotein substrates.	2.14
Prelamin-A/C	LMNA	P02545	LMNA	Component of the nuclear lamina, a fibrous layer on the nucleoplasmic side of the inner nuclear membrane, which provides a framework for the nuclear envelope. The structural integrity of the lamina is strictly controlled by the cell cycle. Lamin A and C are present in equal amounts in the lamina of mammals. Role in nuclear assembly, chromatin organization, nuclear membrane, and telomere dynamics.	2.00
Protein disulfide-isomerase A6	PDIA6	Q15084	PDIA6	Inhibition of misfolded proteins aggregation. Catalysis of the rearrangement of -S-S- bonds in proteins.	1.87
Downregulated					
Cellular retinoic acid-binding protein 2	RABP2	P`29373	CRABP2	Transportation of retinoic acid to the nucleus. Regulation of the access of retinoic acid to the nuclear retinoic acid receptors.	− 10.97
14-3-3 protein zeta/delta	1433Z	P63104	YWHAZ	Idem 14-3-3 protein theta.	− 5.21
Triosephosphate isomerase	TPIS	P60174	TPI1	Role in the gluconeogenesis pathway. Participation in step 1 of the subpathway that synthesizes D-glyceraldehyde 3-phosphate from glycerone phosphate.	− 4.60
Glucose-6-phosphate 1-dehydrogenase	G6PD	P11413	G6PD	Catalysis of the rate-limiting step of the oxidative pentose-phosphate pathway, a route for the dissimilation of carbohydrates besides glycolysis. Provision of reducing power (NADPH) and pentose phosphates for fatty acid/nucleic acid synthesis.	− 4.54
Heat shock protein HSP 90-alpha	HS90A	P07900	HSP90AA1	Idem Heat shock protein HSP 90-beta.	− 4.32
Gamma-glutamylcyclo transferase	GGCT	O75223	GGCT	Catalysis of the formation of 5-oxoproline from gamma-glutamyl dipeptides. Role in glutathione homeostasis. Induction of the release of cytochrome c from mitochondria with resultant induction of apoptosis.	− 4.20
Peroxiredoxin-2	PRDX2	P32119	PRDX2	Thiol-specific peroxidase. Catalysis of the reduction of hydrogen peroxide and organic hydroperoxides to water and alcohols, respectively. Role in cell protection against oxidative stress by detoxifying peroxides and as a sensor of hydrogen peroxide-mediated signaling events. Participation in the signaling cascades of growth factors and tumor necrosis factor-alpha by regulating the intracellular concentrations of H_2_O_2_.	− 4.20
Cathepsin D	CATD	P07339	CTSD	Acid protease active in intracellular protein breakdown. Role in APP processing following cleavage and activation by ADAM30, which leads to APP degradation.	− 3.57
Annexin A4	ANXA4	P09525	ANXA4	Calcium/phospholipid-binding protein which promotes membrane fusion and is involved in exocytosis.	− 3.56
Protein S100-A11	S10AB	P31949	S100A11	Promotion of keratinocytes differentiation and cornification.	− 3.06
Fructose-1,6-bisphosphatase 1	F16P1	P09467	FBP1	Hydrolysis of fructose 1,6-bisphosphate to fructose 6-phosphate in the presence of divalent cations. Rate-limiting enzyme in gluconeogenesis. Regulation of glucose sensing and insulin secretion of pancreatic beta-cells. Modulation of glycerol gluconeogenesis in liver. Regulator of appetite and adiposity.	− 3.00
Adenine phosphoribosyl transferase	APT	P07741	APRT	Catalysis of a salvage reaction resulting in the formation of AMP, that is energically less costly than de novo synthesis. Involved in step 1 of the subpathway that synthesizes AMP from adenine.	− 2.71
Transitional endoplasmic reticulum ATPase	TERA	P55072	VCP	Participation in the fragmentation of Golgi stacks during mitosis and in their reassembly after mitosis. Role in the endoplasmic reticulum (ER) stress-induced pre-emptive quality control, which attenuates the translocation of newly synthesized proteins into the ER and reroutes them to the cytosol for proteasomal degradation. Participation in DNA damage response. Role in the maturation of ubiquitin-containing autophagosomes and the clearance of ubiquitinated protein by autophagy.	− 2.62
Enoyl-CoA delta isomerase 1, mitochondrial	ECI1	P42126	ECI1	Isomerization of 3-cis and trans double bonds into the 2-trans form in a range of enoyl-CoA species. Participation in the fatty acid beta-oxidation pathway, which is part of lipid metabolism.	− 2.54
Actin, cytoplasmic 1	ACTB	P60709	ACTB	Role in various types of cell motility. Ubiquitously expressed in all eukaryotic cells. There are three main groups of actin isoforms in vertebrates. The alpha actins are found in muscle tissues and are a major constituent of the contractile apparatus. The beta and gamma actins coexist in most cell types as components of the cytoskeleton and as mediators of internal cell motility.	− 2.20
Nucleoside diphosphate kinase B	NDKB	P22392	NME2	Role in the synthesis of nucleoside triphosphates other than ATP. Negative regulation of Rho activity. Transcriptional activator of the MYC gene. Binding of DNA in a non-specifically way. Histidine protein kinase activity.	− 2.11
Chloride intracellular channel protein 1	CLIC1	O00299	CLIC1	Capacity of insertion into membranes and formation of chloride ion channels. Membrane insertion seems to be redox-regulated and may occur only under oxydizing conditions. Channel activity depends on the pH. Participation in cell cycle regulation.	− 2.00
Heterogeneous nuclear ribonucleoprotein H	HNRH1	P31943	HNRNPH1	Component of the heterogeneous nuclear ribonucleoprotein (hnRNP) complexes which provide the substrate for the processing events that pre-mRNAs undergo before becoming functional, translatable mRNAs in the cytoplasm. Regulation of pre-mRNA alternative splicing.	− 1.94
Growth factor receptor-bound protein 2	GRB2	P62993	GRB2	Adapter protein that provides a critical link between cell surface growth factor receptors and the Ras signaling pathway.	− 1.94
Superoxide dismutase [Cu-Zn]	SODC	P00441	SOD1	Destruction of radicals which are normally produced within the cells and are toxic to biological systems. Zinc binding promotes dimerization and stabilizes the native form of the protein.	− 1.85
Glucose-6-phosphate isomerase	G6PI	P06744	GPI	Glycolytic enzyme. Participation in step 2 of the subpathway that synthesizes d-glyceraldehyde 3-phosphate and glycerone phosphate from d-glucose. Mammalian GPI can also function as a tumor-secreted cytokine and an angiogenic factor that stimulates endothelial cell motility.	−1.84
Proteasome subunit alpha type-6	PSA6	P60900	PSMA6	Component of the 20S core proteasome complex involved in the proteolytic degradation of most intracellular proteins. Associated with two 19S regulatory particles, forms the 26S proteasome and participates in the ATP-dependent degradation of ubiquitinated proteins. The 26S proteasome plays a role in the maintenance of protein homeostasis by removing misfolded or damaged proteins that could impair cellular functions, as well as proteins whose functions are no longer required.	− 1.82
Phosphoma nnomutase 2	PMM2	O15305	PMM2	Participation in the synthesis of the GDP-mannose and dolichol-phosphate-mannose required for a number of critical mannosyl transfer reactions.	− 1.82
Phospho glycerate mutase 1	PGAM1	P18669	PGAM1	Interconversion of 3- and 2-phosphoglycerate with 2,3-bisphosphoglycerate as the primer of the reaction.	− 1.82
Glycerol-3-phosphate dehydrogenase, mitochondrial	GPDM	P43304	GPD2	Participation in step 1 of the subpathway that synthesizes glycerone phosphate from sn-glycerol 3-phosphate (anaerobic route). Calcium-binding enhance its activity.	− 1.77
Heat shock 70 kDa protein 1A	HS71A	P0DMV8	HSPA1A	Molecular chaperone. Participation in protection of the proteome from stress, folding and transport of newly synthesized polypeptides, activation of proteolysis of misfolded proteins, and formation and dissociation of protein complexes. ATPase activity. Regulation of centrosome integrity during mitosis. Negative regulation of the heat shock-induced HSF1 transcriptional activity during the attenuation and recovery phase period of the heat shock response.	− 1.77
Heat shock 70 kDa protein 1B	HS71B	P0DMV9	HSPA1B	Molecular chaperone. Participation in protection of the proteome from stress, folding and transport of newly synthesized polypeptides, activation of proteolysis of misfolded proteins, and formation and dissociation of protein complexes. ATPase activity. Regulation of centrosome integrity during mitosis.	− 1.77
NADP-dependent malic enzyme	MAOX	P48163	ME1	Catalysis of the reversible oxidative decarboxylation of malate. Generation of NADPH for fatty acid biosynthesis. Link between the glycolytic and citric acid cycles.	− 1.77
6-phosphogluconolactonase	6PGL	O95336	PGLS	Hydrolysis of 6-phosphogluconolactone to 6-phosphogluconate. Participation in step 2 of the subpathway that synthesizes d-ribulose 5-phosphate from d-glucose 6-phosphate (oxidative stage of the pentose phosphate pathway).	− 1.77
Endoplasmic reticulum resident protein 29	ERP29	P30040	ERP29	Role in the processing of secretory proteins within the ER, possibly by participating in the folding of proteins.	− 1.76
Fructose-bisphosphate aldolase A	ALDOA	P04075	ALDOA	Role in glycolysis and gluconeogenesis. It may also function as scaffolding protein. In vertebrates, three forms of this ubiquitous glycolytic enzyme are found: aldolase A in muscle, aldolase B in liver, and aldolase C in brain.	− 1.76
Proteasome subunit beta type-3	PSB3	P49720	PSMB3	Idem Proteasome subunit alpha type-6.	− 1.75
Stress-70 protein, mitochondrial	GRP75	P38646	HSPA9	Chaperone. Role in mitochondrial iron-sulfur cluster (ISC) biogenesis. Regulation of erythropoiesis via stabilization of ISC assembly. It may play a role in the control of cell proliferation and cellular aging.	− 1.72
Peroxiredoxin-6	PRDX6	P30041	PRDX6	Thiol-specific peroxidase that catalyzes the reduction of hydrogen peroxide and organic hydroperoxides to water and alcohols, respectively. It also has phospholipase activity and can reduce short-chain organic, fatty acid, and phospholipid hydroperoxides. Role in cell protection against oxidative stress by detoxifying peroxides and in phospholipid homeostasis.	− 1.70
Heterogeneous nuclear ribonucleo protein F	HNRPF	P52597	HNRNPF	Component of the heterogeneous nuclear ribonucleoprotein (hnRNP) complexes which provide the substrate for the processing events that pre-mRNAs undergo before becoming functional, translatable mRNAs in the cytoplasm. Regulation of alternative splicing events. It binds G-rich sequences in pre-mRNAs and keeps target RNA in an unfolded state.	− 1.70
Actin, aortic smooth muscle	ACTA	P62736	ACTA2	Idem Actin, cytoplasmic 1.	− 1.70
Echinoderm microtubule-associated protein-like 2	EMAL2	O95834	EML2	Tubulin binding protein. Inhibition of microtubule nucleation and growth, resulting in shorter microtubules.	− 1.61
Acylamino-acid-releasing enzyme	ACPH	P13798	APEH	Catalysis of the hydrolysis of the N-terminal peptide bond of an N-acetylated peptide to generate an N-acetylated amino acid and a peptide with a free N-terminus. Preferentially cleavage of Ac-Ala, Ac-Met and Ac-Ser.	− 1.57
Rab GDP dissociation inhibitor beta	GDIB	P50395	GDI2	Regulation of the GDP/GTP exchange reaction of most Rab proteins by inhibiting the dissociation of GDP from them, and the subsequent binding of GTP to them.	− 1.51
Hypoxanthine-guanine phosphoribosyl transferase	HPRT	P00492	HPRT1	Conversion of guanine to guanosine monophosphate, and hypoxanthine to inosine monophosphate. Transference of the 5-phosphoribosyl group from 5-phosphoribosylpyrophosphate onto the purine. Role in the generation of purine nucleotides through the purine salvage pathway.	− 1.51

List of the 50 proteins for which changes in the expression levels were found in MCF7Ecadvar cells relative to MCF7pcDNA3 using the 2D-DIGE and MS technologies. Proteins were listed in a descending order according to their fold change value (1.5-fold change cutoff, ANOVA ≤ 0.005). The protein name and symbol, the Uniprot number, the symbol of the gene that encodes each protein, and a brief description of the protein function are also included in the table. *NADPH* nicotinamide adenine dinucleotide phosphate, *GTP* guanosine triphosphate, *tRNA* transfer RNA, *GDP guanosine* diphosphate, *G*-*rich* guanine-rich, *N*- *amino*, *Ac*- N-protected amino acids, *Ala* alanine, *Met* methionine, *Ser* serine

### Biological characterization of the proteomic analysis results

**Fig. 1:**
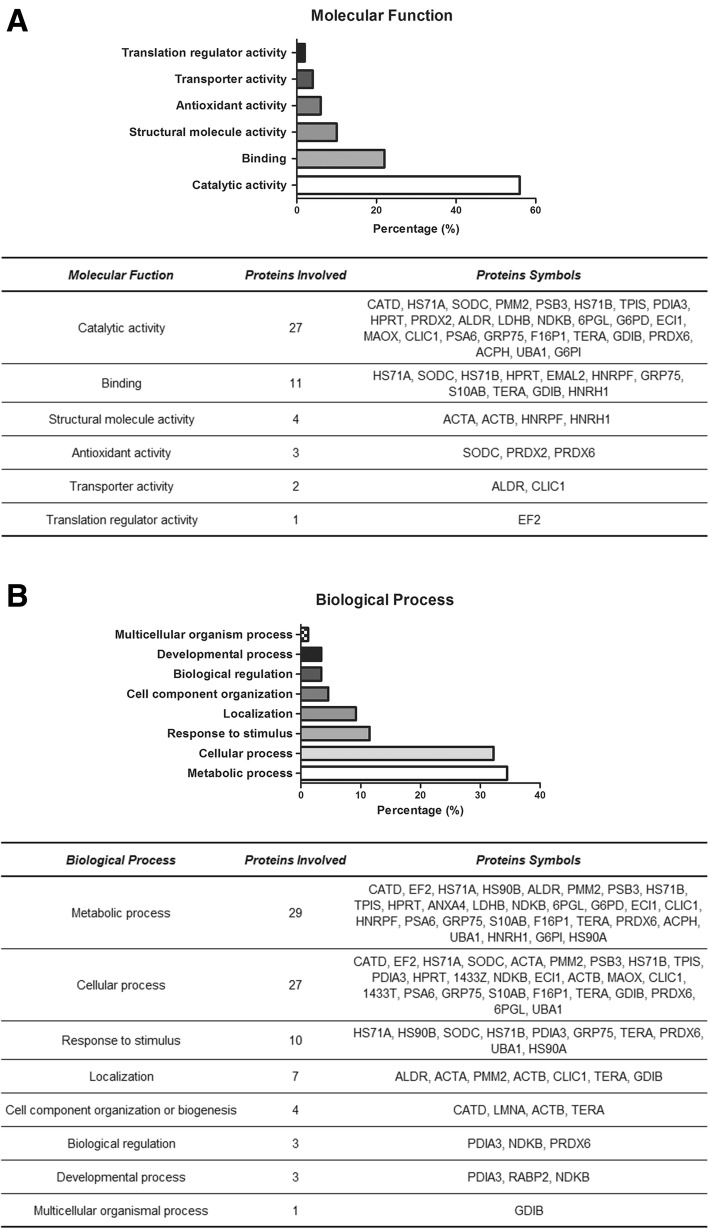
Molecular functions and biological processes associated with the 50 proteins identified. Results obtained with PANTHER. **a** Column graph bar in which the percentage (%) of representation of each molecular function was determined from the number of proteins included in each category (catalytic activity 56.0%, binding 22.0%, structural molecule activity 10.0%, antioxidant activity 6.0%, transporter activity 4.0%, and translation regulator activity 2.0%). A table including the number and symbol of the proteins involved in each molecular function is also shown. **b** Column graph bar in which the percentage (%) of representation of each biological process was determined from the number of proteins included in each category (metabolic process 34.5%, cellular process 32.2%, response to stimulus 11.5%, localization 9.2%, cell component organization or biogenesis 4.6%, biological regulation 3.4%, developmental process 3.4%, and multicellular organism process 1.2%). A table including the number and symbol of the proteins involved in each biological process is also shown

To analyze biological characteristics of the 50 differentially expressed proteins found in MCF7Ecadvar cells, a set of bioinformatics tools were applied. Firstly, proteins were classified using the Protein ANalysis THrough Evolutionary Relationships (PANTHER) tool, by means of their molecular function (Fig. 1a) and the biological processes (Fig. 1b) in which they were involved. As result, catalytic activity was the most represented molecular function (56.0%; 27/50 proteins). Other categories listed were binding, structural molecule, antioxidant activity, transporter, and translation regulator. The energy metabolism was identified as the most affected biological process (34.5%), followed by cellular process (32.2%).

Additional evaluations with the PANTHER tool included cellular component and protein class analysis; results are listed in Additional file 2: Figure S2. With regard to the cellular component classification, the cell part category represented a 52.5% (23/50). Among the protein class categories, 21 (SODC, PRDX2, ALDR, LDHB, 6PGL, G6PD, ECI1, MAOX, PRDX6, CATD, EF2, PSB3, PSA6, F16P1, ACPH, PMM2, TPIS, HPRT, G6PI, GDIB, and UBA1) of the 50 differentially expressed proteins in MCF7Ecadvar cells were included in the six enzymatic categories listed in the protein class classification.

The ingenuity pathway analysis (IPA) bioinformatics tool was run to evaluate the ten canonical pathways most affected in the MCF7Ecadvar cellular model; results were in agreement with the PANTHER analysis (Table 2).

**Table 2:** Ingenuity canonical pathways associated with the proteins identified as differentially expressed between MCF7Ecadvar and MCF7pcDNA3 cells. Results obtained with the IPA tool

Ingenuity canonical pathways	-log(*p* value)	Proteins involved
Glycolysis	0.0836	G6PI, TPIS, PGAM1, F16P1, ALDOA
Gluconeogenesis	0.0827	G6PI, PGAM1, F16P1, ALDOA, MAOX
Protein ubiquitination pathway	0.0535	PSB3, PSA6, HS90B, GRP75, HS71A, HS71B, HS90A, UBA1
14–3-3-mediated signaling	0.0486	1433 T, GRB2, PDIA3, 1433Z, VIME
p70S6K signaling	0.0483	1433 T, GRB2, PDIA3, EF2, 1433Z
PI3K/AKT signaling	0.0478	1433 T, HS90B, GRB2, 1433Z, HS90A
Pentose phosphate pathway (oxidative branch)	0.0439	6PGL, G6PD
Aldosterone signaling in epithelial cells	0.0431	HS90B, PDIA3, GRP75, HS71A, HS71B, HS90A
PPARα/RXRα activation	0.0413	HS90B, GRB2, GPDM, PDIA3, HS90A
Glucocorticoid receptor signaling	0.0410	HS90B, GRB2, ACTB, GRP75, HS71A, HS71B, HS90A

List of the ten canonical pathways most altered in MCF7Ecadvar cells compared with MCF7pcDNA3. They were ordered in a descending form according to the number of the negative of the logarithm of the p value assigned to each of them. The symbol of the proteins identified with differential expression levels between both cell lines that were involved in each pathway are also indicated in the table. *PPARα* peroxisome proliferator-activated receiver α, *RXRα* retinoid X receiver α

In particular, the most altered pathways listed in IPA were glycolysis and gluconeogenesis, two metabolic processes composed by a set of consecutive enzymatic reactions. The pentose phosphate metabolic pathway, an alternative branch of glycolysis to produce sugars that make up DNA and RNA, was also listed in the seventh position. In third position, the protein ubiquitination pathway was found, involving 8/50 proteins identified in the MCF7Ecadvar cell model. This pathway regulates degradation of cellular proteins by the ubiquitin-proteasome system, controlling protein’s half-life and expression levels as well as key cellular processes [13]. The p70S6 kinase (p70S6K) signaling pathway (fifth position) is an important regulator of the cell cycle, which activation requires phosphatidylinositol-4,5-bisphosphate 3-kinase (PI3K)/RAC-alpha serine/threonine-protein kinase (AKT)-dependent signal(s), a pathway found in sixth position [14]. Furthermore, key cellular functions are regulated by the other four canonical pathways identified by the IPA tool, such as cell growth (pathways number 4 and 10), ion transport (pathways number 4 and 8), inflammation, immunity, and metabolism (pathways number 4, 9, and 10) [15–19].

Using IPA, four networks were generated with the 50 proteins differentially expressed in the MCF7Ecadvar cell line. The score associated to each network, the number of molecules among the 50 identified by 2D-DIGE and MS that were part of them (in bold in the second column), and the main diseases and functions associated to each of them are detailed in Table 3.

**Table 3:** Description of the connection networks generated from the proteins identified as differentially expressed between MCF7Ecadvar and MCF7pcDNA3 cells. Results obtained with the IPA tool

Network	Molecules in the network	Score	Molecules involved	Top diseases and functions
1	**ACTA2**, **ACTB**, AIP, C8orf44-SGK3, **CRABP2**, ESR1, GLIPR2, GORASP2, GP9, GP1BA, **GPI**, GRB2, HIST1H2BC, **HNRNPH1**, HSD17B4, **HSP90AA1**, **HSP90AB1**, **HSPA9**, **HSPA1A**, **HSPA1B**, IRS4, LMNB1, **LMNA**, NISCH, PIK3R1, **PDIA3**, REV1, RPS2, SLC6A4, STIP1, TUBA1B, **VCP**, **VIM**, **YWHAQ**, **YWHAZ**	31	16	Connective tissue disorders, developmental disorder, hematological disease
2	AGTR1, **ALDOA**, **APRT**, AXL, AZGP1, SRC, CCND1, **CLIC1**, CTNNB1, **EEF2**, EGFR, EGLN1, ERBB2, **ERP29**, **FBP1**, FGF7, HIF1A, **HNRNPF**, **HNRNPH1**, **HPRT1**, KDM4B, **LDHB**, **LMNA**, LOXL2, MAPK1, MMP7, NCOA3, OGT, **PRDX2**, **PRDX6**, **PSMA6**, **PSMB3**, SMARCA4, WIPF1, **YWHAZ**	31	16	Cardiovascular system development and function, organismal development, cancer
3	**AKR1B1**, AKT1, **ANXA4**, APP, ATP13A2, BAG6, BCL2, BIRC3, CARM1, CDKN1A, CLCA2, CSE1L, CST3, **CTSD**, CUL4B, ESRRG, **G6PD**, IDE, LBR, **ME1**, MEMO1, MYC, NME1, **NME2**, **PGAM1**, PKG, PRL, **S100A11**, SMYD3, **SOD1**, SRSF3, STRAP, TP53, **TPI1**, **UBA1**	19	11	Cellular development, cellular growth and proliferation, free radical scavenging
4	**GPD2**, TNF	2	1	Molecular transport, nucleic acid metabolism, small molecule biochemistry

List of the networks ordered in a descending form according to the score assigned to each of them. The symbol of molecules involved in each of the four networks, the number of molecules among the 50 identified with differential expression levels between MCF7Ecadvar and MCF7pcDNA that were part of each network (in bold in the second column), as well as top diseases and functions associated to each one, are also indicated in the table.

**Fig. 2:**
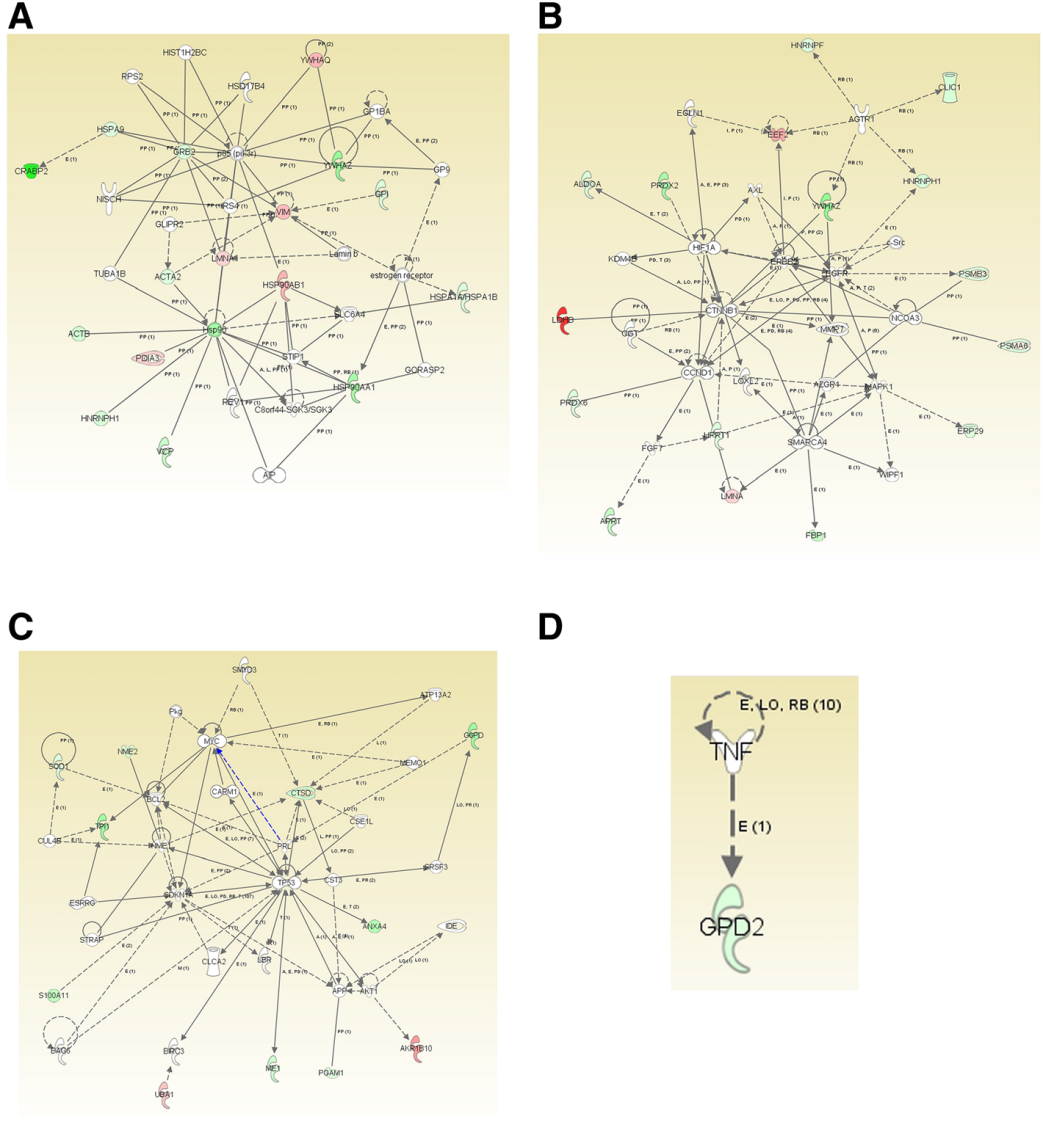
Graphical representation of the connection networks generated from the 50 proteins identified. Results obtained with IPA. In networks **a** 1, **b** 2, **c** 3, and **d** 4, the symbols of the proteins that are part of the list of the 50 identified by 2D-DIGE and MS have a specific color, whereas the ones incorporated by the IPA program as connectors are indicated in white. The function of each of the molecules is also represented by a specific form. For example, in network 1, the NISCH symbol corresponds to cytokine or growth factor, REV1 to enzyme, estrogen receptor to group or complex, PDIA3 to peptidase, AIP to transcription factor, CRABP2 to transporter, TUBA1B to another. In network 3, the symbol assigned to CLCA2 means ionic channel, the one of AKT1 corresponds to kinase, ESRRG to ligand dependent on nuclear receptor, and PGAM1 to phosphatase. Considering the type of connection, the full line indicates direct interaction between two molecules and the dotted line refers to an indirect type of interaction; the direction of the arrows indicates the direction of functionality. The numbers in parentheses located over each connector indicate the amount of scientific works that corroborate the association between these two molecules and the letters specify the type of union through which they interact, such as A: activation, E: expression (includes metabolism and synthesis of chemicals), L: proteolysis (includes degradation of chemicals), LO: location, M: biochemical modification, P: phosphorylation/dephosphorylation, PD: protein-DNA binding, PP: protein-protein binding, PR: protein-RNA binding, RB: regulation of the union, T: transcription

Among the diseases associated to one of the two networks described with the highest score (network number 2) was the term “Cancer.” On the other hand, networks 3 and 4 were related with cellular processes usually altered during tumorigenesis. A graphical representation of each network was also generated with IPA and shown in Fig. 2, in which the type of connection between molecules and their function is indicated.

### Association between proteomic analysis results and BC disease

In order to determine whether the proteins identified as differentially expressed in MCF7Ecadvar cells have already been reported to be associated with BC, the DisGeNET bioinformatics tool was used. A comprehensive study was conducted, in which all databases available in DisGeNET (curated, predicted, and literature) were considered to search for all the genes associated with terms that include the words “Breast” or “Mammary”: “Carcinoma,” “Cancer,” “Neoplasms,” or “Tumorigenesis”. In total, 36 terms were identified (March 24, 2018) and, from the gene lists associated with each of these terms, a list of 5261 genes (Additional file 3: Figure S3) was generated using the Venny 2.1.0 interactive tool. A total of 39 from the 50 identified genes had already been reported associated with these terms. Using the DisGeNET tool, it was also found that for ten of the 11 genes not previously related to the 36 analyzed terms, an altered expression had been reported in other carcinomas (Table 4).

**Table 4:** Diseases associated with the no common molecules between BC genes found in DisGeNET and proteins identified as differentially expressed between MCF7Ecadvar and MCF7pcDNA3 cells. Results obtained with the DisGeNET tool

Gene	Diseases
ECI1	Noninfiltrating intraductal carcinoma
PSMA6	Multiple myeloma, osteosarcoma
PMM2	Adenocarcinoma of lung (disorder)
GPD2	Prostatic neoplasms
HSPA9	Liver neoplasms, secondary malignant neoplasms of liver, liver and intrahepatic biliary tract carcinoma, renal cell carcinoma, leukemia, myeloid leukemia, colorectal carcinoma, colon carcinoma, prostate carcinoma, carcinoma of lung, stomach carcinoma, central neuroblastoma, medullary carcinoma of thyroid, adenocarcinoma, carcinogenesis, neoplasm metastasis, recurrent tumor
HNRNPF	Colorectal neoplasms, glioma, colon carcinoma
EML2	Melanoma, pancreatic carcinoma, Lewis lung malignant neoplasm of pancreas, carcinoma, tumor-associated vasculature
GDI2	Squamous cell carcinoma of esophagus
PDIA3	Liver carcinoma, stomach neoplasms, prostate carcinoma, squamous cell carcinoma, leukemia myelocytic acute, ovarian carcinoma, epithelial ovarian carcinoma, secondary malignant neoplasm of lymph node, malignant neoplasm of larynx, melanoma, cervical adenocarcinoma, secondary malignant neoplasm of lung, adenocarcinoma, carcinogenesis
UBA1	Squamous cell carcinoma, neoplasm metastasis

List of the 11 genes from the 50 that coded for the proteins found with different expression levels between MCF7Ecadvar and MCF7pcDNA3 for which no association was found with the 36 DisGeNET terms that included the words “Breast” or “Mammary,” and “Carcinoma,” “Cancer,” “Neoplasms,” or “Tumorigenesis.” The gene symbol and the diseases in which each of them has been reported altered are indicated. The PSMB3 gene was not found in DisGeNET

Finally, the PSMB3 gene was not found in the DisGeNET database, but a manual search performed in PubMed led to the identification of one scientific paper that related PSMB3 with BC [20], and two reports with colorectal cancer and multiple myeloma [21, 22].

### Validation studies of 2-DIGE and MS proteomic analysis results

**Fig. 3:**
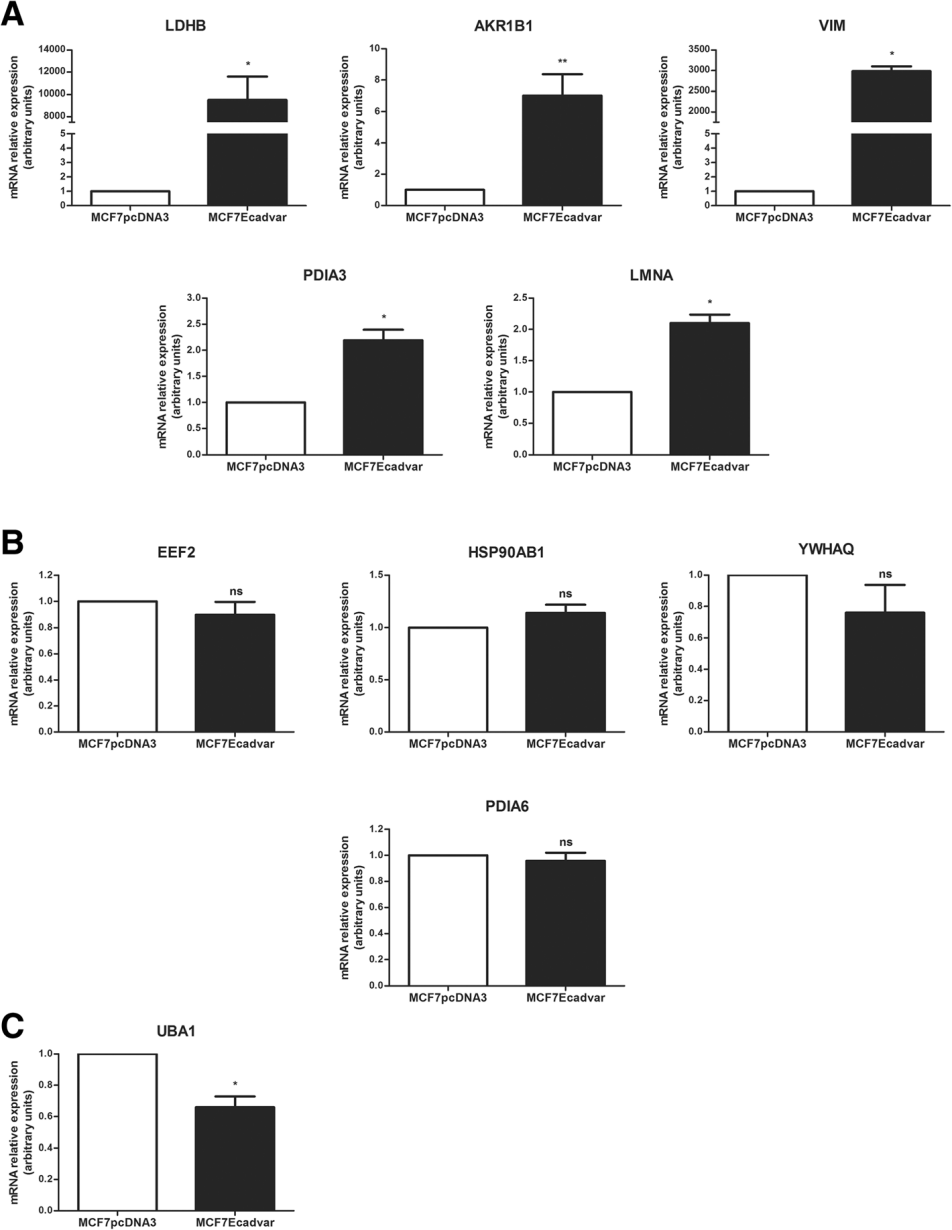
Transcripts expression analysis of the ten most upregulated molecules in MCF7Ecadvar cells relative to MCF7pcDNA3. Quantitative real-time PCR of **a** LDHB, AKR1B1, VIM, PDIA3, LMNA; **b** EEF2, HSP90AB1, YWHAQ, PDIA6; and **c** UBA1 in MCF7pcDNA3 and MCF7Ecadvar cells. The relative expression was calculated as described in the “Materials and Methods” section, using GAPDH as the endogenous gene and the MCF7pcDNA3 cell line as reference. *p < 0.05, **p < 0.01, *ns* not significant

**Fig. 4:**
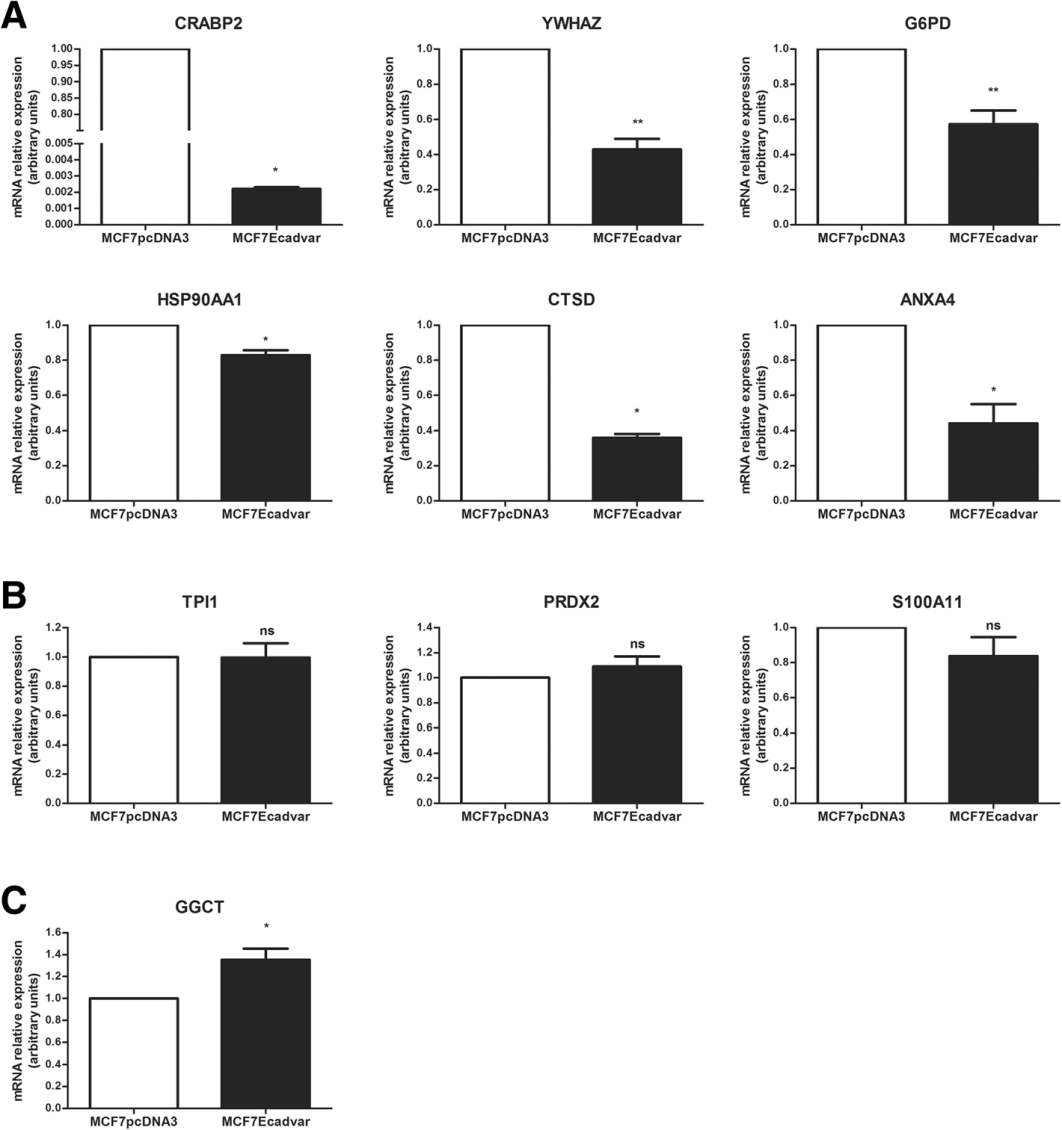
Transcripts expression analysis of the ten most downregulated molecules in MCF7Ecadvar cells relative to MCF7pcDNA3. Quantitative real-time PCR of **a** CRABP2, YWHAZ, G6PD, HSP90AA1, CTSD, ANXA4; **b** TPI1, PRDX2, S100A11; and **c** GGCT in MCF7pcDNA3 and MCF7Ecadvar cells. The relative expression was calculated as described in the “Materials and Methods” section, using GAPDH as the endogenous gene and the MCF7pcDNA3 cell line as reference. *p < 0.05, **p < 0.01, *ns* not significant

Validation of the results obtained by the 2D-DIGE and MS technologies was done at the mRNA level, by quantitative real-time PCR. Specific primers were designed for the genes encoding the top 10 up- and downregulated proteins in MCF7Ecadvar cells relative to MCF7pcDNA3 (Additional file 4: Figure S4). Among the ten upregulated proteins, the same trend to an increased expression found at the protein level was determined for LDHB, AKR1B1, VIM, PDIA3, and LMNA transcripts in MCF7Ecadvar cells compared with MCF7pcDNA3 (Fig. 3a). On the other hand, no significant differences were observed in the expression levels of the EEF2, HSP90AB1, YWHAQ, and PDIA6 mRNAs between both cell lines (Fig. 3b). For the UBA1 protein-coding transcript, a significant decreased expression (*p* < 0.05) was registered in MCF7Ecadvar cells relative to the control cell line, contrasting with the increased protein level found by proteomics (Fig. 3c). Among the top 10 downregulated proteins, a decreased mRNA expression was also found in CRABP2, YWHAZ, G6PD, HSP90AA1, CTSD, and ANXA4 (Fig. 4a), while TPI1, PRDX2, and S100A11 transcript levels did not show significant differences between MCF7Ecadvar and MCF7pcDNA3 cells (Fig. 4b). Finally, an increased expression of the GGCT mRNA was found in MCF7Ecadvar cells compared to the control, results that contrasted with the decreased protein expression levels registered in the proteomic analysis (Fig. 4c).

### LDHB expression analysis

Among the 50 proteins identified as differentially expressed in MCF7Ecadvar cells, the LDHB enzyme showed the highest level of increase (Fold = 9.34; Table 1). LDHB is a cytoplasmic protein that catalyzes the interconversion of pyruvate and lactate, with the concomitant interconversion of nicotinamide adenine dinucleotide (reduced form; NADH) and nicotinamide adenine dinucleotide (oxidized form; NAD^+^). Previous reports have associated changes in LDHB expression with tumor progression. However, while decreased LDHB levels were reported in prostate, bladder, and hepatocellular carcinomas [23–25], an increased LDHB expression was described in lung and pancreatic adenocarcinomas, non-small-cell lung cancer and osteosarcoma [26–29]. In BC, its expression has been reported in different cell lines and in human tumors [30, 31].

**Fig. 5:**
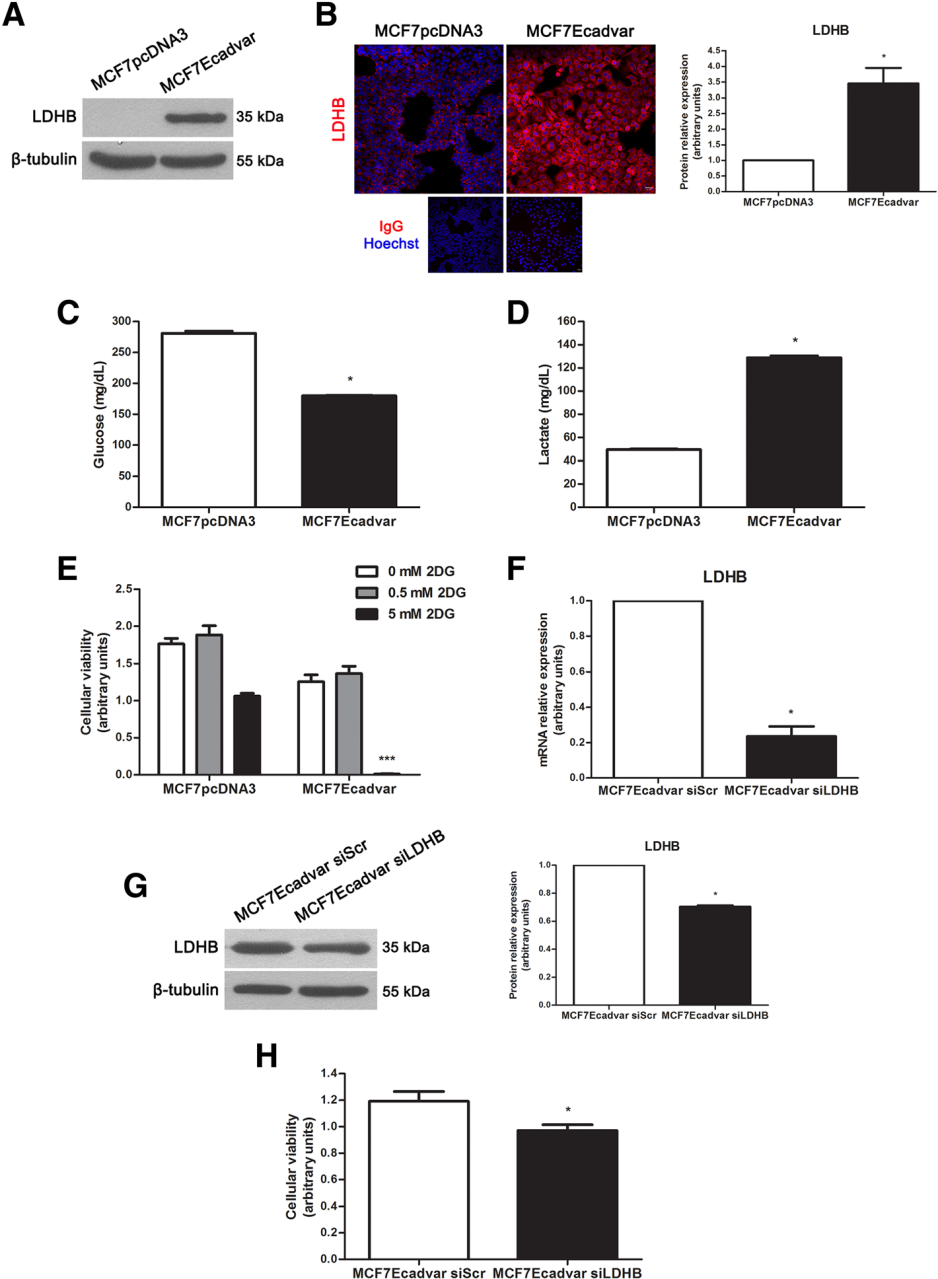
Expression analysis of LDHB in MCF7Ecadvar and MCF7pcDNA3 cells. **a** Western immunoblotting of the 35 kDa LDHB form in total protein extracts of MCF7pcDNA3 and MCF7Ecadvar cells. The immunodetection of β-tubulin (55 kDa) was used as loaded control. **b** Immunofluorescence analysis of LDHB (× 200 magnification, scale bar 50 μm) coupled to nuclear staining with dye Hoechst 33342 in MCF7pcDNA3 and MCF7Ecadvar cells. Negative controls were included for both cell lines. Quantification of the LDHB specific signal was also done and indicated in the bar graph. **c**, **d** Determination of **c** glucose and **d** lactate concentrations (mg/dL) in the 72-h-conditioned media of MCF7Ecadvar and MCF7pcDNA3 culture cells. Measurements were done with a Cobas 8000 equipment. **e** Cellular viability analysis of MCF7pcDNA3 and MCF7Ecadvar culture cells after 24 h treatment with 0, 0.5, or 5 mM of the 2DG glycolytic inhibitor. **f** Quantitative analysis of LDHB by real-time PCR in MCF7Ecadvar siScr and MCF7Ecadvar siLDHB cells. The relative expression was calculated as described in the “Materials and Methods” section, using GAPDH as the endogenous gene and the MCF7Ecadvar siScr cell line as reference. **g** Western immunoblotting of the 35 kDa LDHB form in total protein extracts of MCF7Ecadvar siScr and MCF7Ecadvar siLDHB cells. The immunodetection of β-tubulin (55 kDa) was used as loaded control. Quantification of the LDHB specific signal was also done and indicated in the bar graph. **h** Cellular viability analysis of MCF7Ecadvar siScr and MCF7Ecadvar siLDHB culture cells after 72 h treatment with 100 pmoL/mL of a LDHB specific siRNA. *p < 0.05, ***p < 0.001

Immunodetection of the LDHB protein by Western immunoblotting revealed, under the conditions evaluated, no detectable levels of the enzyme in total protein extracts of MCF7pcDNA3 cells, whereas a 35 kDa signal was observed for the MCF7Ecadvar cell line (Fig. 5a). By immunofluorescence microscopy, a specific signal localized at the cytoplasm of MCF7pcDNA3 and MCF7Ecadvar cells was detected, which was 3.45 times higher in MCF7Ecadvar than in control cells (Fig. 5b).

Different types of cancers, including BC, show high rates of glucose consumption associated with lactate production, even in the presence of normal levels of oxygen, a phenomenon known as aerobic glycolysis or Warburg effect [32, 33]. When glucose concentration (mg/dL) was measured in the conditioned media of MCF7Ecadvar and MCF7pcDNA3 cell lines, a significant decrease (*p* < 0.05) was observed in the former relative to the control (Fig. 5c). The assessment of the lactate concentration revealed higher levels (*p* < 0.05) of this metabolite in the conditioned medium of MCF7Ecadvar cells than in the control cell medium (Fig. 5d). In agreement with these findings, a significant increase (*p* < 0.05) in the expression of the monocarboxylate transporters 1 (MCT1) and 4 (MCT4) transcripts was determined in MCF7Ecadvar cells compared with MCF7pcDNA3 (Additional file 5: Figure S5).

In order to evaluate whether the metabolic differences observed between MCF7Ecadvar and MCF7pcDNA3 cells affected cellular viability, cultures of both cell lines were incubated with the glycolytic inhibitor 2-deoxy-d-glucose (2DG), a modified-glucose molecule (substitution of hydroxyl group for hydrogen at the second carbon atom) that blocks glycolysis by inhibiting hexokinase activity, impairs cancer cell growth, and induces cell death through apoptosis [34]. Incubation of MCF7Ecadvar cells with 5 mM 2DG led to a decrease (*p* < 0.001) in cell viability but not in control cells. Contrasting, the addition of 0.5 mM 2DG did not induce significant changes in MCF7Ecadvar cell viability compared with the control, suggesting a concentration-dependent effect of the inhibitor (Fig. 5e). Finally, the association between LDHB expression and MCF7Ecadvar cell proliferation was evaluated by transfecting cells with a LDHB specific small interfering RNA (siRNA). As shown in Fig. 5f, g, a decreased expression of the transcript and protein of LDHB was found in MCF7Ecadvar cells treated with 100 pmoL/mL of LDHB siRNA relative to the MCF7Ecadvar cells transfected with a scramble siRNA (siScr) used as negative control after 72 h of treatment. This negative modulation of LDHB expression resulted in a significant reduction (*p* < 0.05) of MCF7Ecadvar cell viability, when compared with results in the MCF7Ecadvar siScr treated cells (Fig. 5h).

### E-cadherin variant and LDHB mRNA expression analysis in tumor tissue samples from BC patients

To translate the results obtained with the MCF7Ecadvar cellular model to the clinic, 21 breast tumor tissues from women who underwent biopsy and/or surgery for BC management before receiving radiotherapy or chemotherapy were analyzed. The histological and molecular characteristics of the samples are detailed in Additional file 6: Table S1.

**Fig. 6:**
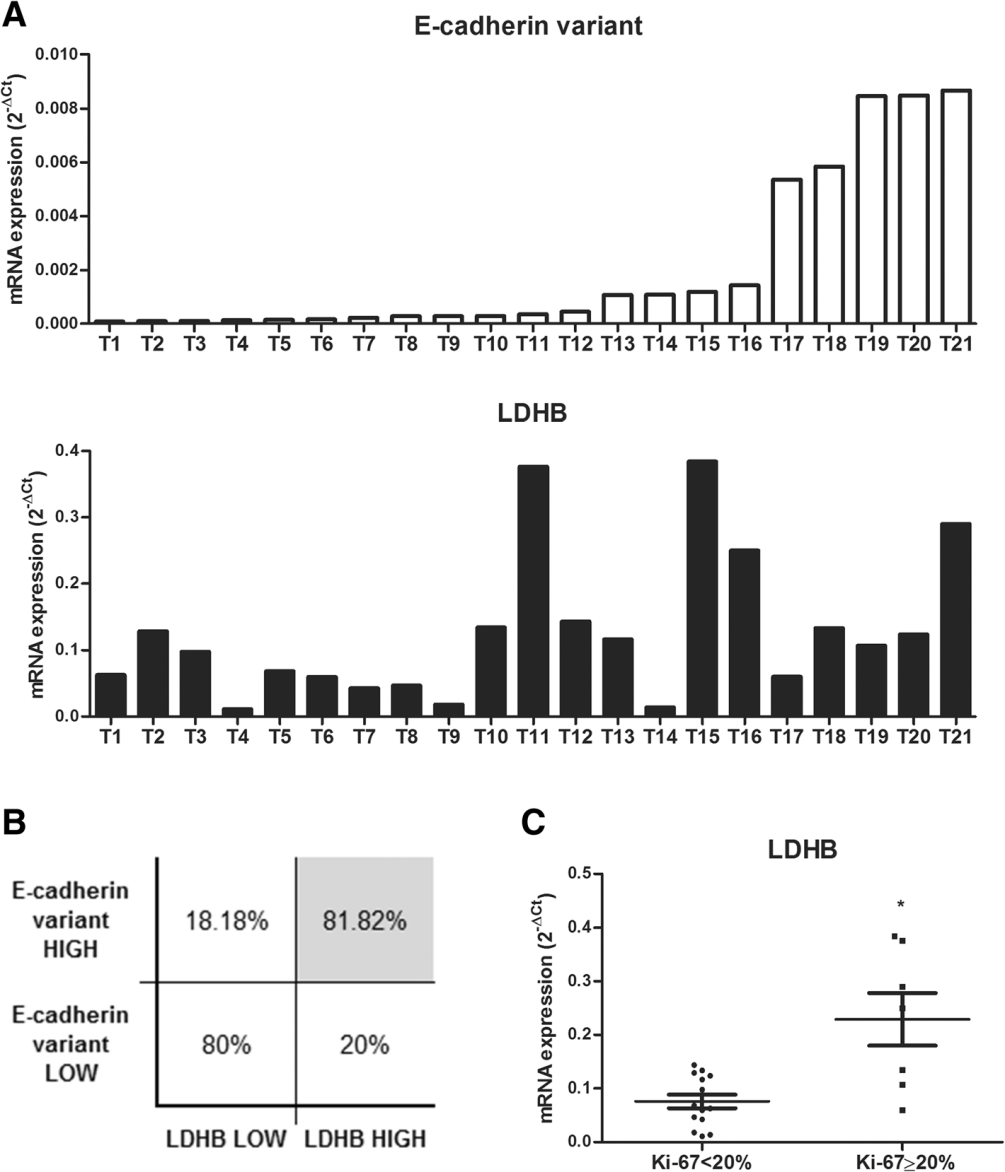
Transcripts expression analysis of E-cadherin variant and LDHB in human breast tumor samples. **a** Quantitative expression analysis of E-cadherin variant and LDHB by real time PCR in 21 human breast tumor tissues. Samples were numbered and ordered in an ascending form according with the 2^-ΔCt^ values obtained for the E-cadherin variant mRNA. **b** Transcripts levels association analysis of E-cadherin variant with LDHB in 21 human breast tumor tissues. The expression levels of both markers were indicated as low or high using the median value of E-cadherin variant and LDHB transcripts levels as cutoff values. The gray box highlights a significant association between E-cadherin variant and LDHB overexpression using the Fisher exact test (*p* < 0.0089). **c** Analysis of the LDHB mRNA expression levels in 21 human breast tumor samples divided in 2 groups according to their Ki-67 labeling index (Ki-67 < 20% and Ki-67 ≥ 20%). *p < 0.05\

Tissues were processed for total RNA extraction and the E-cadherin variant and LDHB transcripts expression levels were evaluated by quantitative real-time PCR. As a result, detectable amounts of both mRNAs were registered in the 21 breast tumors analyzed (Fig. 6a and Additional file 7: Table S2), although expression levels of each transcript differed among tumors.

Samples were numbered and organized in ascending order according to the 2^-ΔCt^ values obtained for the E-cadherin variant transcript, which ranged from 0.000078 to 0.008669. For the LDHB mRNA, 0.011010 was the lowest 2^-ΔCt^ value registered and 0.384302 the highest. Using the median value of E-cadherin variant and LDHB transcripts levels (0.000353 and 0.1073, respectively) as cutoff values to group samples in “low expression” or “high expression” for each molecule, a 2 × 2 contingency table was made and the Fisher’s exact test was applied. This analysis revealed a significant association (odds ratio 0.06, confidence interval 0.01–0.40, *p* < 0.0089) between the expression levels of the E-cadherin splice variant and LDHB transcripts. As shown in Fig. 6b, 80% of the breast tumor samples with low E-cadherin variant mRNA expression showed low levels of LDHB mRNA, while 81.82% of the samples with high E-cadherin variant expression levels presented high LDHB mRNA expression.

Finally, taking into account that an increased expression of LDHB has been associated with a high cellular viability and proliferation in previous reports [28, 29], and considering that this behavior was also observed in the MCF7Ecadvar cellular model (Fig. 5h), LDHB mRNA levels were compared in the 21 breast tumor tissues grouped according to the Ki-67 labeling index (samples with Ki-67 < 20% and samples with Ki-67 ≥ 20%). The immunohistochemical analysis of Ki-67 is regularly utilized to evaluate tumor cell proliferation, and is considered a prognostic and predictive marker in BC [35]. As result of this analysis, breast tumor tissues from the Ki-67 ≥ 20% group showed higher LDHB transcript expression levels than those from the Ki-67 < 20% group.

## Discussion

In the mammary gland, E-cadherin is one of the main cellular adhesion molecules required for morphogenesis and maintenance of the epithelial structure as well as for epithelial cell survival [36]. BC is a complex disease that involves cell dedifferentiation and proliferation of the mammary epithelium, leading to tissue disorganization. Deregulation of E-cadherin expression has been associated with BC progression and aggressiveness, and the underlying mechanisms involved in E-cadherin expression changes have been extensively studied [37, 38]. Dr. Vazquez-Levin’s group identified a novel human E-cadherin splice variant transcript that differs from the E-cadherin wt mRNA in 34 bp deleted in exon 14. Stable transfection of the variant transcript in MCF-7 cells showed a detrimental effect over the E-cadherin wt expression, and additional molecular changes characteristic of the EMT process and of an aggressive cellular behavior [12]. Based on this background information, a global protein analysis was carried out using the 2D-DIGE and MS technologies to unleash the molecules and mechanisms of the cellular alterations observed associated with the overexpression of the E-cadherin variant in MCF-7 cells.

In the 2D-DIGE technique, protein mixtures of test and control samples are obtained, labeled with different fluorescent dyes, mixed, and subjected to 2D-electrophoresis, by which protein components are resolved based on their isoelectric point and apparent molecular weight. The inclusion of an internal standard containing all the proteins of the samples under study reduces gel-to-gel variations, allows for individual experiments normalization, and confers a high sensitivity and reproducibility for differential quantitative analysis of protein expression between experimental conditions [39]. Despite having some limitations, such as under-representation of hydrophobic, membrane, and low abundance proteins, and the difficulty to resolve proteins with extreme isoelectric points or with very high or low molecular weight, the comparative 2D-DIGE method has been applied in biological and biomedical research areas to discover novel biomarkers, improve diagnostics and prognostics, and for therapy-monitoring. The potential of this approach lies in an accurate detection and quantification of protein isoforms, giving information that can be lost when using standard immunochemical assays and genomic analysis for protein detection [39, 40]. This electrophoresis procedure is followed by protein spot digestion and MS analysis, allowing peptide identification with high specificity and sensitivity. The 2D-DIGE technique coupled to MS protein identification was previously applied by members of the team [41], as well as by others, for the characterization of the global proteome of diverse experimental models. In particular, this approach was used for the assessing of EMT protein profiles [42–45]. In this regard, Vergara and collaborators [46] carried out a stable knockdown of E-cadherin wt expression in MCF-7 cells by transfecting with an shRNA, and changes in the proteomic signature were evaluated using a 2D-gel electrophoresis and MS approach, also observing changes in the expression of proteins involved in carbohydrate metabolism, such as PGAM1, also identified in this study, but with the opposite trend.

The proteomic study done on MCF7Ecadvar and MCF7pcDNA3 cells led to the identification of 50 proteins depicting a significant differential expression level between cell lines, which are involved in several signaling pathways and participate in the regulation of a variety of important cellular processes. In particular, 29 of the 50 proteins identified were associated with cell metabolism, and the glycolysis and gluconeogenesis metabolic pathways were listed as the most altered in the MCF7Ecadvar cellular model. Other processes categorized as response to stimulus, localization and organization of cellular components, biological regulation and development, were also listed. Among the 50 proteins, there were found enzymes (LDHB, ALDR, APT, TERA, HPRT, UBA1, G6PD, ACPH, NDKB, 6PGL, F16P1, G6PI, PGAM1, ECI1, ALDOA, MAOX, GGCT, PMM2, GPDM, TPIS, SODC, PRDX2, and PRDX6), proteases (CATD, PSB3, and PSA6), binding (ANXA4 and EMAL2) and transport (RABP2 and CLIC1) molecules, chaperones (HS90A, HS90B, HS71A, HS71B, GRP75, PDIA3, PDIA6, ERP29), structural molecules (ACTB, ACTA, VIME and LMNA), adapter/regulatory molecules (1433Z, 1433 T, GRB2, GDIB and EF2), splicing (HNRH1 and HNRPF), and differentiation (S10AB) factors. Transcript expression analysis showed the same trend of change for 11 (LDHB, AKRIB1, VIM, PDIA3, LMNA, CRABP2, YWHAZ, G6PD, HSP90AA1, CTSD, and ANXA4) of the 20 molecules evaluated in MCF7Ecadvar compared with MCF7pcDNA3 cells. Interestingly, the specific knockdown of E-cadherin wt was also associated with expression changes in a significant number of metabolic proteins and the catalytic activity was one of the most altered molecular function [46], suggesting that some of the molecular changes triggered by E-cadherin variant mRNA overexpression in MCF-7 could be associated with its role as a negative E-cadherin wt modulator. Part of the expression changes recorded for the molecules differentially expressed in the MCF7Ecadvar cell line were reported in samples from patients with different cancer types, in particular with BC. In some cases, these changes have also been associated with an aggressive tumor phenotype and a worse patient prognosis.

In the last decade, it was demonstrated that tumor cells exhibit an altered expression of different metabolic enzymes and metabolite transporters to increase the generation of energy necessary for a high proliferation and migration, as well as for survival and invasion [47]. Glycolysis is a critical catabolic process through which a glucose molecule is cleaved to produce two molecules of pyruvate, two molecules of adenosine triphosphate (ATP), and two of NADH. In normal cells, the fate of pyruvate depends, to a large extent, on oxygen availability. In its presence, pyruvate is oxidized to acetyl coenzyme A (acetil-CoA), which is then completely oxidized to carbon dioxide and water in the tricarboxylic acid cycle, generating 3 NADH and 1 flavin adenine dinucleotide hydroquinone form (FADH_2_). As a result, the reducing power produced through these processes is used to yield large amounts of energy in the form of ATP through the mitochondrial oxidative phosphorylation system. In contrast, in the absence of oxygen, glycolysis is decoupled from oxidative phosphorylation and pyruvate is transformed into lactate through the anaerobic glycolysis metabolic pathway, becoming the primary source of ATP production.

Almost 100 years ago, Warburg and collaborators reported that aerobic glycolysis and lactate generation were increased in tumor cells, allowing a faster production of energy than through the oxidative phosphorylation carried out by normal cells [48]. Among the ten proteins upregulated in the MCF7Ecadvar cellular model, LDHB showed the highest positive fold change. Since LDHB catalyzes interconversion of pyruvate (the glycolysis final product) and lactate, quantification of glucose and lactate levels in MCF7Ecadvar and MCF7pcDNA3 conditioned media was done. These studies revealed an increased glucose consumption and lactate production by MCF7Ecadvar cells. Moreover, the decrease in the expression of enzymes involved in gluconeogenesis (FBP1, GPI, PGAM1, ALDOA, ME1, and TPI1) and in the pentose phosphate pathway (G6PD and PGLS) registered in MCF7Ecadvar cells with respect to control would contribute to the increased availability of glucose-6-phosphate for the aerobic glycolysis over the pathways responsible of the synthesis of glycogen, fatty acids, and nucleic acids. An association between E-cadherin variant and LDHB levels was also demonstrated through the analysis of both transcripts in 21 human breast tumor samples.

To achieve the reprogramming of metabolism, tumor cells depend on the action of specific transcription factors, such as hypoxia-inducible factor 1-alpha (HIF1A) and MYC [49–51]. The activation of the PI3K, AKT, mTOR, Ras, Raf, Src, and EGFR oncogenes [52, 53], as well as the loss of tumor suppressor genes like PTEN and TP53 [54, 55], were also demonstrated to exacerbate tumor cell growth and metabolism. Some of these molecules were involved in the list of the ten canonical pathways identified by the IPA tool as the most altered in MCF7Ecadvar compared with MCF7pcDNA3 cells (mTOR, PI3K, and AKT) or were included as connectors into the IPA networks to link some of the proteomic molecules (EGFR, HIF1A, AKT1, MYC, and TP53). Particularly, it was reported that LDHB is targeted by the mTOR complexes and plays a fundamental role in the tumorigenesis triggered by the PI3K/AKT signaling pathway [56], all major mediators of cell survival. These findings could be related to the diminished cellular viability found in MCF7Ecadvar cells after the specific knockdown of LDHB expression. Moreover, the analysis of LDHB mRNA levels in 21 human breast tumor tissues showed that samples with Ki-67 labeling index ≥ 20% had higher levels of the LDHB transcript than those with a Ki-67 < 20%, reinforcing that the overexpression of the LDHB enzyme is necessary for a faster tumor growth [30, 31] and a more aggressive tumoral behavior [57, 58].

## Conclusions

Taken together, results obtained from the 2D-DIGE and MS proteomic analysis done in MCF7Ecadvar and MCF7pcDNA3 cells revealed the complexity of the molecular effects associated with the overexpression of the novel E-cadherin splice variant mRNA in MCF-7 cells. While a large proportion (39 of 50) of the proteins identified as differentially expressed between both cell lines had been previously reported in the literature associated with BC, for some of them the available information is scarce and sometimes contradictory. Thus, the stable transfection of the novel E-cadherin variant transcript in MCF-7 cells may represent a useful experimental model to deepen in the knowledge of the cellular alterations associated with the deregulation of E-cadherin wt in this complex and heterogeneous disease. Specifically, this is the first report that describes the involvement of a novel E-cadherin splice variant in triggering molecular changes related to BC progression and aggressiveness and its relationship with LDHB expression.

## Methods

### Chemicals

Chemicals were of analytical or tissue culture grade and purchased from Sigma-Aldrich (Sigma; St. Louis, MO, USA). Molecular biology and electrophoresis reagents were purchased from Invitrogen (Thermo Fisher Scientific; Waltham, MA, USA), Qiagen (Hilden, Germany), and Bio-Rad (Hercules, CA, USA), unless specified.

The following antibodies were used: anti E-cadherin (610,181, mouse, monoclonal; Becton Dickinson Biosciences [BD], San Diego, CA, USA), anti LDHB (431.1, mouse, monoclonal; Santa Cruz Biotechnology [SCBT], Santa Cruz, CA, USA), and anti β-tubulin (clone D66, mouse, monoclonal; Sigma). For immunocytochemistry, a Cy3-labeled anti-mouse (Sigma) IgG was used as secondary antibody. An anti-mouse (Vector Laboratories Inc.; Burlingame, CA, USA) IgG coupled to horseradish peroxidase was employed as secondary antibody in Western immunoblotting assays.

### In silico analysis

The following bioinformatics tools were used in this study:

#### PANTHER

Comprehensive software system for classifying proteins (and their genes) depending on the family or subfamily they belong, the molecular function they have, the biological process they are involved, or the way in which they participate on it. The classifications result from human data healing and sophisticated bioinformatics algorithms [59, 60].

#### DisGeNET

Discovery platform that integrates information on gene-disease associations by text-mining approaches from expert-curated databases and scientific literature. Data is organized according to their type and level of curation in curated (gene-disease associations from UNIPROT and CTD human databases), predicted (CTD mouse and rat data, RGD and MGD), literature (GAD, LHGDN, and BeFree), and all (curated, predicted, and literature). The gene-disease associations can be ranked according to a 0 to 1 DisGeNET score and are annotated with the DisGeNET gene-disease association type ontology. The score takes into account the number and type of sources (i.e., level of curation and organisms), as well as the number of publications supporting the association. It can be accessed through the web interface [61] or using a Cytoscape plugin [62] (see below).

#### Venny

Online tool to compare lists of genes or proteins by performing Venn diagrams. It also allows visualization of specific elements of each list, as well as common elements between them [63].

#### IPA

Web-based software application for the analysis, integration, and interpretation of data derived from omics experiments. It also transforms a list of genes or gene products into a set of networks based on the ingenuity knowledge base, which has been abstracted into a large network called global molecular network composed of thousands of genes that interact with each other. The relationship between two genes is called connection and two genes are considered to be connected if there is a path between them in the network, i.e., a set of genes connecting one to the other. If the relationship between genes of a list of interest is not direct, the IPA program relates them through other neighboring genes present in the global molecular network. In addition, IPA assigns a score value to each network and the higher the score the higher the degree of confidence for the connections identified in the network [64].

### Laboratory procedures

All procedures reported in this study were done following protocols that complied with biosafety and ethical guidelines accepted worldwide.

### Tissue samples

Twenty-one tumor tissue samples from patients diagnosed with BC of different subtypes and stages of the disease were obtained from the Hospital Italiano of Buenos Aires and the Instituto de Oncología Ángel H. Roffo, Buenos Aires, Argentina. Sample collection protocols were approved by the institutional Ethics Committees and patient informed consents were obtained in all cases. The study was conducted in accordance with the principles of the Declaration of Helsinki. Samples were from women older than 18 years old, who underwent biopsy and/or surgery because of BC diagnosis. The breast tumor tissues of patients that received radiotherapy or chemotherapy before surgery were excluded from the study.

### Cell cultures

Cell lines were grown in appropriate culture conditions as previously described [12]. Briefly, MCF-7 human BC cells, as well as MCF7Ecadvar and MCF7pcDNA3 stably transfected cells, were cultured in sterile culture dishes (Nunc, Thermo Fisher Scientific) at 37 °C in a humid atmosphere containing 5% CO_2_ in air. Cells were grown in the Dulbecco’s modified Eagle’s medium Ham’s Nutrient Mixture F-12 (DMEM F-12; Gibco, Thermo Fisher Scientific) cell culture medium, supplemented with 10% fetal calf serum (FBS), 100 U/mL penicillin, 100 mg/mL streptomycin, and 2 mM glutamine. Upon reaching 80% confluence, cells were sub-cultured using a 0.25% sterile solution of trypsin and 0.025% ethylenediaminetetraacetic acid (EDTA) in phosphate-buffered saline (PBS).

### Proteomic analysis

Proteomic analysis was done employing 2D-DIGE technology coupled to protein identification by MS at the Proteomics Service of the Vall d’Hebron Institute of Oncology, Vall d’Hebron University Hospital, Barcelona, Spain, basically as previously reported [65].

For sample preparation, triplicates of MCF7Ecadvar and MCF7pcDNA3 cell cultures were grown in Petri dishes (Nunc, Thermo Fisher Scientific) as described before. When cells reached 60–70% confluence, the growth medium was replaced by medium without FBS supplementation. After 24 h, cultures were collected with 5 mM EDTA, centrifuged 5 min at 200×*g* and stored at − 70 °C until use.

For the proteomic analysis, cellular pellets were lysed with 400 μL of DIGE lysis buffer (pH 8.5; 30 mM Tris, 7 M urea, 2 M thiourea, 4% CHAPS) to achieve protein extraction. After sonication, protein extracts were purified using the 2D-CleanUp kit (GE Healthcare; Buckinghamshire, UK) and re-dissolved in DIGE lysis buffer. Protein concentration was determined with the Bio-Rad RC DC Protein Assay (Bio-Rad). Proteins were then labeled using the Ettan DIGE method (GE Healthcare). A pool consisting of equal amounts of each of the samples analyzed in this study was prepared to be used as an internal standard for quantitative comparisons. Triplicates from MCF7Ecadvar and MCF7pcDNA3 cell cultures were alternatively labeled with Cy3 and Cy5 cyanine dyes to avoid possible bias by labeling efficiency, and the internal standard was labeled with the Cy2 dye. The recommended marker/protein ratio of 400 picomoles of marker per 50 μg of total proteins was used.

MCF7Ecadvar and MCF7pcDNA3 total protein samples were separated in a first-dimension by the IEF technique using the IPGphor system (GE Healthcare). The IPGphor strips have a length of 24 cm and cover a range of 3 to 10 pH units. Isoelectrical focusing was carried out overnight or until reaching 8000 Vh. Total proteins separation in a second-dimension was carried out by laying the strips on 12.5% isocratic Laemmli gels (24 × 20 cm) on an Ettan DALTsix Electrophoresis System (GE Healthcare). Gels were run at 20 °C at constant power (2.5 W) per gel for 30 min, followed by 17 W per gel until the bromophenol blue tracking front reached the end of the gel. When the run was finished, gels were scanned with the Typhoon FLA 7000 Scanner (GE Healthcare; Cy2: 488/520 nm, Cy3: 532/580 nm and Cy5: 633/670 nm excitation/emission wavelengths). Image analysis and statistical quantification of relative protein abundances were performed using Progenesis Samespots (Nonlinear Dynamics, UK) and a DeCyder 6.0 version software (GE Healthcare). Protein spots that met a 1.5-fold change cutoff and an ANOVA ≤ 0.005 were considered. Once detected, the spots differentially expressed between MCF7Ecadvar and MCF7pcDNA3 samples were excised from the gels using the Ettan SpotPicker System (GE Healthcare). In-gel trypsin digestion was performed as previously described [66] using autolysis stabilized trypsin (Promega, Madison, WI, USA) and tryptic digests were purified with ZipTip Pipette Tips (EMD Millipore, Cork, Ireland). Spectra of the protein spots selected were obtained by the matrix-assisted laser desorption ionization-time of flight (MALDI-TOF), MALDI/TOF-TOF, and liquid chromatography-electrospray ionization-MS in tandem (LC-ESI-MS/MS) techniques. Protein identity determination in each spot recovered was done through the Mascot Database Search Program.

### Transfection with LDHB siRNA

To knockdown endogenous LDHB gene expression, 250,000 MCF7Ecadvar cells per well were seeded in 6-well plates (Nunc, Thermo Fisher Scientific). After 24 h, cells were transfected with 100 pmoL/mL of a specific LDHB or a scramble (SCBT) siRNA, using Lipofectamine 2000 (Thermo Fisher Scientific) and following manufacturer’s instructions. Seventy-two hours after transfection, cells were recovered with EDTA 5 mM to perform LDHB mRNA and protein expression analysis. In addition, assessment of cellular viability was done with the CellTiter 96 AQueous Non-Radioactive Cell Proliferation Assay (Promega; Madison, WI, USA). Briefly, three replicates of 5000 cells were done from both LDHB and control siRNA transfections, and cultured for 5 days at 37 °C in 5%CO_2_ in air. Cells were harvested and each replicate was seeded in 5-wells of a 96-well plate. Then, 20 μL of the reagent solution of 2 mL MTS [3-(4,5-dimethylthiazol-2-yl)-5-(3-carboxymethoxyphenyl)-2-(4-sulfophenyl)-2H-tetrazolium] and 0.1 mL PMS (phenazine methosulfate) were added to each well and incubated for 1 h, after which absorbance was read at 490 nm on a microtiter spectrophotometer plate reader (Thermo Fisher Scientific). Controls without cells were included for background estimation.

### Total RNA extraction and real-time PCR procedures

Total RNA from MCF-7, MCF7pcDNA3, and MCF7Ecadvar cell cultures, as well as from breast tumor tissue samples, was isolated with the Trizol reagent (Thermo Fisher Scientific) following the manufacturer’s instructions and quantified (Nanodrop, Thermo Fisher Scientific). Synthesis of complementary DNA (cDNA) was performed with oligo-dT and SuperScript™ III Reverse Transcriptase (Thermo Fisher Scientific). Negative controls omitting the RNA or the reverse transcriptase enzyme were included in all cases. Standard PCR amplification protocols were carried out using the *Taq*I DNA polymerase (Qiagen). Reverse transcription procedures were verified by amplification of a glyceraldehyde 3-phosphate dehydrogenase (GAPDH) cDNA fragment.

Quantitative assessment of gene expression was done by real-time PCR with the Bio-Rad Real Time PCR unit using the SYBR Select Master Mix (Thermo Fisher Scientific). All samples were run in triplicates and negative controls (no template) were included in all cases. Primers used in this study were designed using the Primer-BLAST program (http://www.ncbi.nlm.nih.gov/tools/primer-blast) and synthesized by Eurofins MWG Operon (Louiswille, KY, USA).

Relative expression was calculated using GAPDH as endogenous control. Expression levels of Ecadvar mRNA were determined with primers originally designed for its identification [12]: forward: 5′-GACCAAGTGACCACCTTAGA-3′, reverse: 5′GACCACCGCTCTCTTAGC3 ′. When indicated, the MCF7pcDNA3 cell line or the MCF7Ecadvar cells transfected with the scramble siRNA (MCF7Ecadvar siScr) were used as references as specifically indicated. The calculation used to describe these relations was 2^-ΔΔCt^, where ΔΔCt = [ΔCt test sample − ΔCt reference sample] and ΔCt = [Ct gene under study − Ct endogenous gene].

### One-dimension protein electrophoresis and Western immunoblotting

For total protein extraction, cell cultures were washed twice at 4 °C with PBS (pH 7.4) supplemented with 2 mM CaCl_2_ and placed at − 20 °C until processing. Cells were then resuspended in Laemmli sample buffer, boiled for 5 min, and sonicated three times for 30 s at maximum power (Sonifier Cell Disruptor, model W-140; Heat Systems-Ultrasonics Inc., Plainview, NY, USA). Cell lysates were centrifuged 30 min at 10,000×*g* at 4 °C to eliminate cellular debris and stored at − 70 °C until used. Protein concentration was determined by the Bradford Protein Assay (Bio-Rad). Protein mixtures of cellular lysates were supplemented with 5% β-mercaptoethanol final concentration, boiled for 10 min, and subjected to SDS-PAGE in 8 or 10% polyacrylamide gels. Molecular weight standards (Bio-Rad) were included in all runs. After Western immunoblotting, a replica of protein patterns was obtained on nitrocellulose membranes (Hybond-ECL; GE Healthcare) using standard procedures. Membranes were placed for 1 h at room temperature with PBS containing 0.02% Tween-20 and 5% skimmed milk powder (blocking buffer), followed by overnight incubation at 4 °C with the specific primary antibody diluted in blocking buffer. Blots were washed, placed for 1 h at room temperature with the secondary antibody in blocking buffer, and developed with enhanced chemiluminescence (ECL kit; GE Healthcare), following the procedure suggested by the manufacturer. Replicates of three experiments were obtained and a densitometric analysis of the bands was performed using the ImageJ software (Wright Cell Imaging Facility, UHNR, CA, USA), when indicated. A representative image of each experiment is shown.

### Fluorescence immunocytochemistry

MCF7Ecadvar and MCF7pcDNA3 cells grown on glass coverslips until 80% confluence were fixed with 4% (*v*/*v*) paraformaldehyde in PBS for 10 min and permeabilized in PBS supplemented with 0.2% (*v*/*v*) Triton X-100. For fluorescence immunocytochemistry, non-specific binding sites were blocked with 4% (*w*/*v*) bovine serum albumin in PBS followed by overnight incubation at 4 °C with an anti LDHB antibody (2 μg/mL) or mouse IgG (control). After several washes, cells were incubated with purified secondary antibody coupled to Cy3-fluorophor for an additional hour. Nuclear staining was done by incubation with Hoechst 33342 (Sigma). Coverslips were mounted with Vectashield anti-fade solution (Vector). Images were acquired with a Nikon laser confocal microscope C1 (Nikon, Tokyo, Japan; excitation lines: 488 nm and 544 nm, emission filters: 515–530 nm and 570-LP nm) using a × 20 objective and were analyzed using the ImageJ software when specified.

### Assessment of glucose and lactate concentrations

Conditioned media of MCF7Ecadvar and MCF7pcDNA3 cell cultures were collected and subjected to assessment of glucose and lactate concentrations (mg/dL). Briefly, cells were grown to 70% confluence as previously described. Cultures were washed twice with PBS and media was replaced by fresh medium supplemented with 2% fetal bovine serum. After 72 h incubation, the media were collected in sterility, centrifuged at 200×*g* for 10 min to remove cellular debris and used. The glucose and lactate concentrations were determined at the Dr. Enrique Rossi Diagnostic Centre, Buenos Aires, Argentina, using a Cobas 8000 equipment (Roche Diagnostics, Basel, Switzerland).

### Cell treatment with 2DG

To evaluate the effect of 2DG (Sigma D6134) upon growth of MCF7Ecadvar and control cell cultures, 5000 cells were seeded in triplicates in a 96-well plate in DMEM medium freed of phenol red containing 5% FBS. After 18–20 h, culture medium was replaced by the same medium containing 2DG at 0.5 mM or 5 mM final concentrations and incubated for 96 h at 37 °C in 5% CO_2_ in air. Cells incubated in the absence of 2DG were also included and considered as the control condition. At the end of the incubation, cell viability was evaluated with the CellTiter 96 AQueous Non-Radioactive Cell Proliferation Assay, adding the reagent and reading the absorbance after 4-h incubation as described above.

### Statistical analysis

All experiments were run in triplicates. Data are presented as the mean ± standard error of the mean (SEM). Evaluations done on MCF7Ecadvar and MCF7pcDNA3 cells were carried out using the Mann-Whitney test or the two-way ANOVA test followed by Bonferroni’s post-hoc test analysis. Studies performed on human samples were done using the Fisher’s exact test or the Mann-Whitney test, as detailed. A *p* < 0.05 was considered significant for each comparison. Statistical analyses and graphs were performed using the GraphPad Prism version 5.01 software (GraphPad Software, La Jolla, CA, USA).

## Additional files

Additional file 1: **Figure S1.** E-cadherin protein expression analysis in MCF7Ecadvar and MCF7pcDNA3 cells. Western immunoblotting of the 120 kDa E-cadherin wild type (E-cadherin wt) full length form in total protein extracts obtained from 3 MCF7pcDNA3 (1 to 3, 10 μg per lane) and 3 MCF7Ecadvar (4 to 6, 20 μg per lane) cell cultures. The immunodetection of β-tubulin (55 kDa) was used as loaded control. (TIF 2449 kb)

Additional file 2: **Figure S2.** Cellular components and protein classes associated with the 50 proteins identified. Results obtained with PANTHER. (A) Column graph bar in which the percentage (%) of each cellular component was determined from the number of proteins included in each category (cell part 52.5%, organelle 26.1%, macromolecular complex 10.9%, extracellular region 6.5% and membrane 4.3%). A table including the number and symbol of the proteins involved in each protein class category is also shown. (B) Column graph bar in which the percentage (%) of each protein class was determined from the number of proteins included in each category (oxidoreductase 19.6%; hydrolase 15.2%; isomerase 10.9%; chaperone 8.7%; transferase 8.7%; cytoskeletal protein 6.5%; nucleic acid binding 6.5%; ligase, enzyme modulator 4.3% and lyase, 4.3% each; calcium-binding protein, membrane-traffic protein signaling molecule, transfer/carrier protein and transporter, 2.2% each). A table including the number and symbol of the proteins involved in each protein class category is also shown. (TIF 2499 kb)

Additional file 3: **Figure S3.** Genes associated with human BC. Results obtained with DisGeNET. List of the 5261 human genes that emerged from the 36 terms (“Breast Carcinoma”, “Female Breast Carcinoma”, “Stage 0 Breast Carcinoma”, “Stage IIIA Breast Carcinoma”, “Stage IIIB Breast Carcinoma”, “Invasive Ductal Breast Carcinoma”, “Invasive Lobular Breast Carcinoma”, “Secretory Breast Carcinoma”, “Inflammatory Breast Carcinoma”, “Adenoid Cystic Breast Carcinoma”, “Apocrine Breast Carcinoma”, “Invasive Apocrine Breast Carcinoma”, “Intermediate Grade Ductal Breast Carcinoma In Situ”, “Breast Carcinoma Metastatic in the Skin”, “Breast Cancer 3”, “Breast Cancer Stage II”, “Stage III Breast Cancer AJCC v6”, “Breast Cancer Recurrent”, “Bilateral Breast Cancer”, “Breast Cancer and Pregnancy”, “Breast Cancer, Familial”, “Breast Cancer (non-specific) Premenopausal”, “Contralateral Breast Cancer”, “Unilateral Breast Neoplasms”, “Malignant Neoplasm of Breast”, “Malignant Neoplasm of Female Breast”, “Malignant Neoplasm of Breast Stage I”, “Malignant Neoplasm of Breast Staging”, “Secondary Malignant Neoplasm of Female Breast”, “Triple Negative Breast Neoplasms”, “Mammary Carcinoma, Human”, “Mammary Ductal Carcinoma”, “Mammary Neoplasms”, “Mammary Neoplasms, Human”, “Mammary Neoplasms, Experimental” and “Mammary Tumorigenesis”) found in DisGeNET containing the words “Breast” or “Mammary”, and “Carcinoma”, “Cancer”, “Neoplasms” or “Tumorigenesis”. The 39 genes in common with those that code for the 50 proteins identified by 2D-DIGE and MS as differentially expressed between the MCF7Ecadvar and MCF7pcDNA3 cell lines are highlighted. (PDF 168 kb)

Additional file 4: **Figure S4.** Specific primers for the top-10 upregulated and downregulated molecules among the 50 identified. List of the primers used for the evaluation of the expression levels of the mRNAs that code for the 10 most (A) upregulated and (B) downregulated proteins identified with differential expression levels between MCF7Ecadvar and MCF7pcDNA3 cells by 2D-DIGE and MS. The sequence of the forward and reverse primers, as well as the size of each amplified product, are indicated. (TIF 2455 kb)

Additional file 5: **Figure S5.** Transcripts expression analysis of MCT1 and MCT4 in MCF7Ecadvar and MCF7pcDNA3 cells. Quantitative expression analysis of (A) MCT1 and (B) MCT4 lactate transporters by real time PCR in MCF7pcDNA3 and MCF7Ecadvar cells. The relative expression was calculated as described in the Materials and Methods section, using GAPDH as the endogenous gene and the MCF7pcDNA3 cell line as reference. *p < 0.05. (TIF 4993 kb)

Additional file 6: **Table S1.** Histological and molecular characteristics of breast tumor tissue samples. Description table in which the histological type and grade, the presence or absence of metastasis, the expression pattern of ER, PR and HER2, the percentage (%) of Ki-67 staining, as well as the molecular subtype of 21 breast tumor tissues, are detailed. (DOCX 17 kb)

Additional file 7: **Table S2.** Transcripts expression analysis of E-cadherin variant and LDHB in human breast tumor samples. Quantitative expression analysis of E-cadherin variant and LDHB by real time PCR in 21 human breast tumor tissues. Samples were numbered and ordered in an ascending form according with the 2^-ΔCt^ values obtained for the E-cadherin variant mRNA. (DOCX 15 kb)

## Abbreviations

μg: Micrograms
2D: Two-dimensional
2D-DIGE: Two-dimensional differential gel electrophoresis
2DG: 2-Deoxy-d-glucose
6PGL: 6-Phosphogluconolactonase
Ac: N-protected amino acids
ACPH: Acylamino-acid-releasing enzyme
ACTA: Actin, aortic smooth muscle
ACTB: Actin, cytoplasmic 1
ADAM10: Disintegrin and metalloproteinase domain-containing protein 10
ADAM17: Disintegrin and metalloproteinase domain-containing protein 17
AKR1B1: Aldose reductase
AKT: RAC-alpha serine/threonine-protein kinase
Ala: Alanine
ALDOA: Fructose-bisphosphate aldolase A
ALDR: Aldose reductase
ANOVA: Analysis of variance
ANXA4: Annexin A4
APT: Adenine phosphoribosyltransferase
AS: Alternative Splicing
ATP: Adenosine triphosphate
BC: Breast cancer
BCL2: Apoptosis regulator Bcl-2
CASP8: Caspase-8
CATD: Cathepsin D
CCND2: G1/S-specific cyclin-D2
CCNG1: Cyclin-G1
CDH1: Epithelial cadherin
cDNA: complementary DNA
CLIC1: Chloride intracellular channel protein 1
CoA: Coenzyme A
CRABP2: Cellular retinoic acid-binding protein 2
CTSD: Cathepsin D
CUL4B: Cullin 4B
DICER1: Endoribonuclease Dicer
dL: Deciliter
E-cadherin wt: Epithelial cadherin wild type
E-cadherin: Epithelial cadherin
ECI1: Enoyl-CoA delta isomerase 1, mitochondrial
EDTA: Ethylenediaminetetraacetic acid
EEF2: Elongation factor 2
EF2: Elongation factor 2
EGF: Epidermal growth factor
EGFR: Epidermal growth factor receptor
EMAL2: Echinoderm microtubule-associated protein-like 2
EMT: Epithelial-mesenchymal transition
ERK: Extracellular signal-regulated kinase
ERP29: Endoplasmic reticulum resident protein 29
FADH_2_: Flavin adenine dinucleotide hydroquinone form
FBS: Fetal calf serum
FOXA1: Hepatocyte nuclear factor 3-alpha
G: Guanine
G6PD: Glucose-6-phosphate 1-dehydrogenase
G6PI: Glucose-6-phosphate isomerase
GAPDH: Glyceraldehyde 3-phosphate dehydrogenase
GDIB: Rab GDP dissociation inhibitor beta
GDP: Guanosine diphosphate
GGCT: Gamma-glutamylcyclotransferase
GLIPR2: Golgi-associated plant pathogenesis-related protein 1
GPDM: Glycerol-3-phosphate dehydrogenase, mitochondrial
GPI: Glucose-6-phosphate isomerase
GRB2: Growth factor receptor-bound protein 2
GRP75: Stress-70 protein, mitochondrial
GTP: Guanosine triphosphate
HIF1A: Hypoxia-inducible factor 1-alpha
HMGA1: High mobility group protein HMG-I/HMG-Y
HMGA2: High mobility group protein HMGI-C
HNRH1: Heterogeneous nuclear ribonucleoprotein H
HNRPF: Heterogeneous nuclear ribonucleoprotein F
HPRT: Hypoxanthine-guanine phosphoribosyltransferase
HS71A: Heat shock 70 kDa protein 1A
HS71B: Heat shock 70 kDa protein 1B
HS90A: Heat shock protein HSP 90-alpha
HS90B: Heat shock protein HSP 90-beta
HSP90AA1: Heat shock protein HSP 90-alpha
HSP90AB1: Heat shock protein HSP 90-beta
IEF: Isoelectric focusing
IGF1R: Insulin-like growth factor 1 receptor
IPA: Ingenuity pathway analysis
kDa: Kilodaltons
LC-ESI-MS/MS: Liquid chromatography-electrospray ionization-ms in tandem
LDHB: Lactate dehydrogenase B chain
LMNA: Prelamin-A/C
MALDI/TOF-TOF: Matrix-assisted laser desorption ionization-time of flight
MAOX: NADP-dependent malic enzyme
MCF7Ecadvar: MCF-7 human breast cancer cells stably transfected with a pcDNA3 eukaryotic plasmid containing an E-cadherin variant mRNA
MCF7pcDNA3: MCF-7 human breast cancer cells stably transfected with a pcDNA3 empty plasmid
MCT1: Monocarboxylate transporter 1
MCT4: Monocarboxylate transporter 4
ME1: NADP-dependent malic enzyme
MEK: Dual specificity mitogen-activated protein kinase
Met: Methionine
mg: Milligrams
miRNA: microRNA
mL: Milliliters
MS: Mass spectrometry
mTOR: Mammalian target of rapamycin
N: Amino
NAD^+^: Nicotinamide adenine dinucleotide, oxidized form
NADH: Nicotinamide adenine dinucleotide, reduced form
NADPH: Nicotinamide adenine dinucleotide phosphate
NDKB: Nucleoside diphosphate kinase B
NF-kappa-B: Nuclear factor kappa-light-chain-enhancer of activated B cells
nm: Newton metro
NMD: Nonsense-Mediated mRNA Decay
p70S6K: p70S6 kinase
PANTHER: Protein ANalysis THrough Evolutionary Relationships
PBS: Phosphate-buffered saline
PCR: Polymerase chain reaction
PDIA3: Protein disulfide-isomerase A3
PDIA6: Protein disulfide-isomerase A6
PGAM1: Phosphoglycerate mutase 1
PGLS: 6-Phosphogluconolactonase
PKC: Protein kinase C
PLCgamma: Phospholipase C gamma
PMM2: Phosphomannomutase 2
pmoL: Picomoles
PMS: Phenazine methosulfate
PPARα: Peroxisome proliferator-activated Receiver α
PRDM1: PR domain zinc finger protein 1
PRDX2: Peroxiredoxin-2
PRDX6: Peroxiredoxin-6
PSA6: Proteasome subunit alpha type-6
PSB3: Proteasome subunit beta type-3
PSMB3: Proteasome subunit beta type-3
RABP2: Cellular retinoic acid-binding protein 2
RING: E3 ubiquitin-protein ligase RING
RXRα: Retinoid X receiver α
S100A11: Protein S100-A11
S10AB: Protein S100-A11
SEM: Standard error of the mean
Ser: Serine
siRNA: small interfering RNA
siScr: siRNA scramble
SODC: Superoxide dismutase [Cu-Zn]
STAT: Signal transducer and activator of transcription
TERA: Transitional endoplasmic reticulum ATPase
TGF: Transforming growth factor
TP53: Cellular tumor antigen p53
TPI1: Triosephosphate isomerase
TPIS: Triosephosphate isomerase
tRNA: Transfer RNA
UBA1: Ubiquitin-like modifier-activating enzyme 1
v/v: Volume/volume
v/w: Volume/weight
VIM: Vimentin
VIME: Vimentin
miR-122-5p: microRNA 122-5p
1433T: 14-3-3 protein theta
1433Z: 14-3-3 protein zeta/delta
YWHAQ: 14-3-3 protein theta
YWHAZ: 14-3-3 protein zeta/delta
F16P1: Fructose-1,6-bisphosphatase 1
FBP1: Fructose-1,6-bisphosphatase 1
PI3K: Phosphatidylinositol-4,5-bisphosphate 3-kinase
MTS: 3-(4,5-dimethylthiazol-2-yl)-5-(3-carboxymethoxyphenyl)-2-(4-sulfophenyl)-2H-tetrazolium
