# Supplementary figures and images for: Characterization of the molecular changes associated with the overexpression of a novel epithelial cadherin splice variant mRNA in a breast cancer model using proteomics and bioinformatics approaches: identification of changes in cell metabolism and an increased expression of lactate dehydrogenase B
Source: Cancer Metab. 2019 May 9;7:5. doi: 10.1186/s40170-019-0196-9 (PMC6507066; doi:10.1186/s40170-019-0196-9)

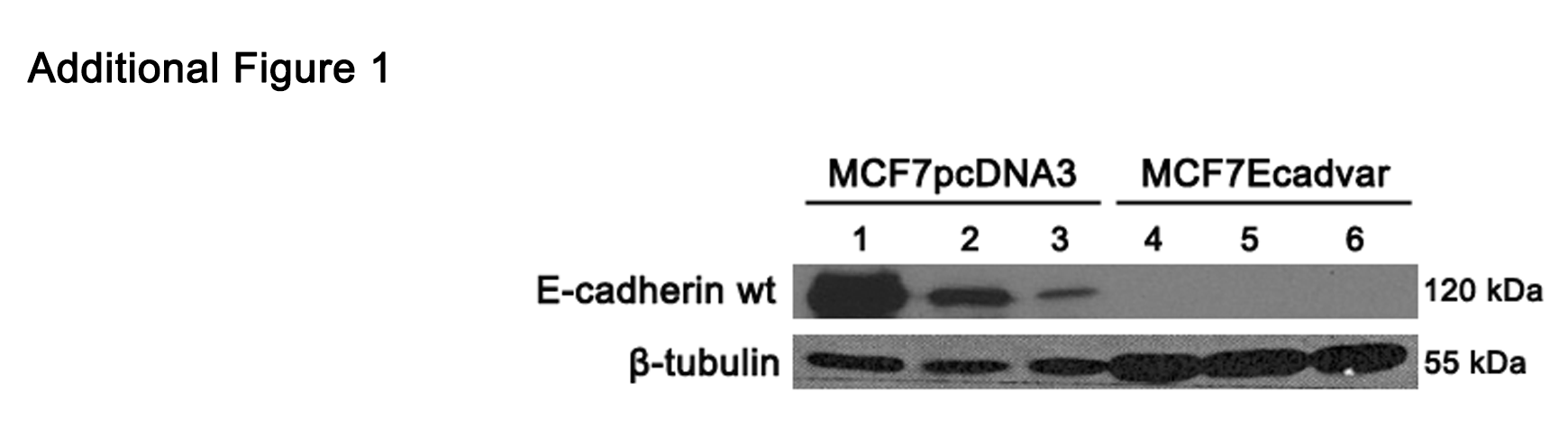

Supplement: Supplementary file 1 — Figure S1. E-cadherin protein expression analysis in MCF7Ecadvar and MCF7pcDNA3 cells. Western immunoblotting of the 120 kDa E-cadherin wild type (E-cadherin wt) full length form in total protein extracts obtained from 3 MCF7pcDNA3 (1 to 3, 10 μg per lane) and 3 MCF7Ecadvar (4 to 6, 20 μg per lane) cell cultures. The immunodetection of β-tubulin (55 kDa) was used as loaded control. (TIF 2449 kb) [file 40170_2019_196_MOESM1_ESM.tif]

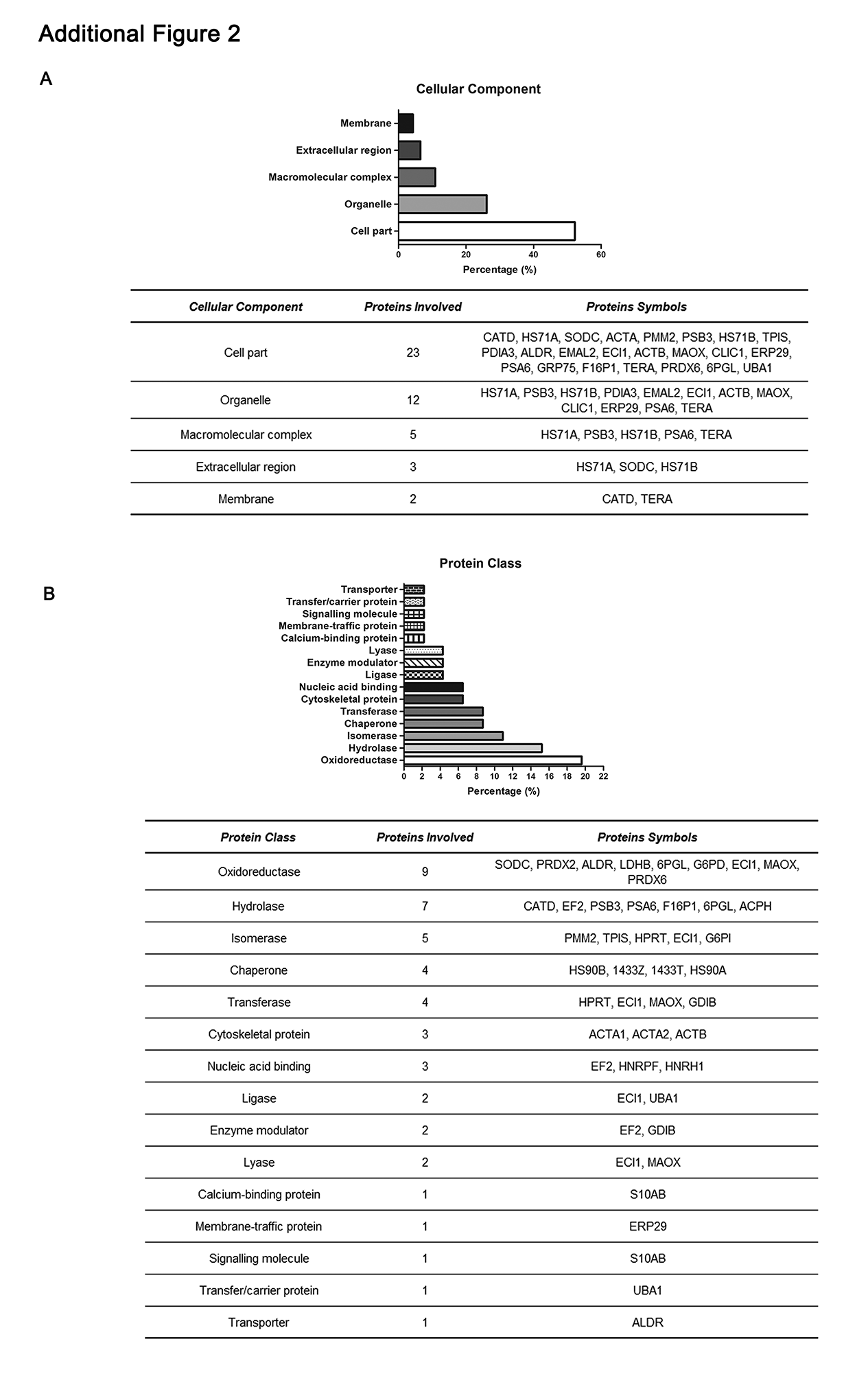

Supplement: Supplementary file 2 — Figure S2. Cellular components and protein classes associated with the 50 proteins identified. Results obtained with PANTHER. (A) Column graph bar in which the percentage (%) of each cellular component was determined from the number of proteins included in each category (cell part 52.5%, organelle 26.1%, macromolecular complex 10.9%, extracellular region 6.5% and membrane 4.3%). A table including the number and symbol of the proteins involved in each protein class category is also shown. (B) Column graph bar in which the percentage (%) of each protein class was determined from the number of proteins included in each category (oxidoreductase 19.6%; hydrolase 15.2%; isomerase 10.9%; chaperone 8.7%; transferase 8.7%; cytoskeletal protein 6.5%; nucleic acid binding 6.5%; ligase, enzyme modulator 4.3% and lyase, 4.3% each; calcium-binding protein, membrane-traffic protein signaling molecule, transfer/carrier protein and transporter, 2.2% each). A table including the number and symbol of the proteins involved in each protein class category is also shown. (TIF 2499 kb) [file 40170_2019_196_MOESM2_ESM.tif]

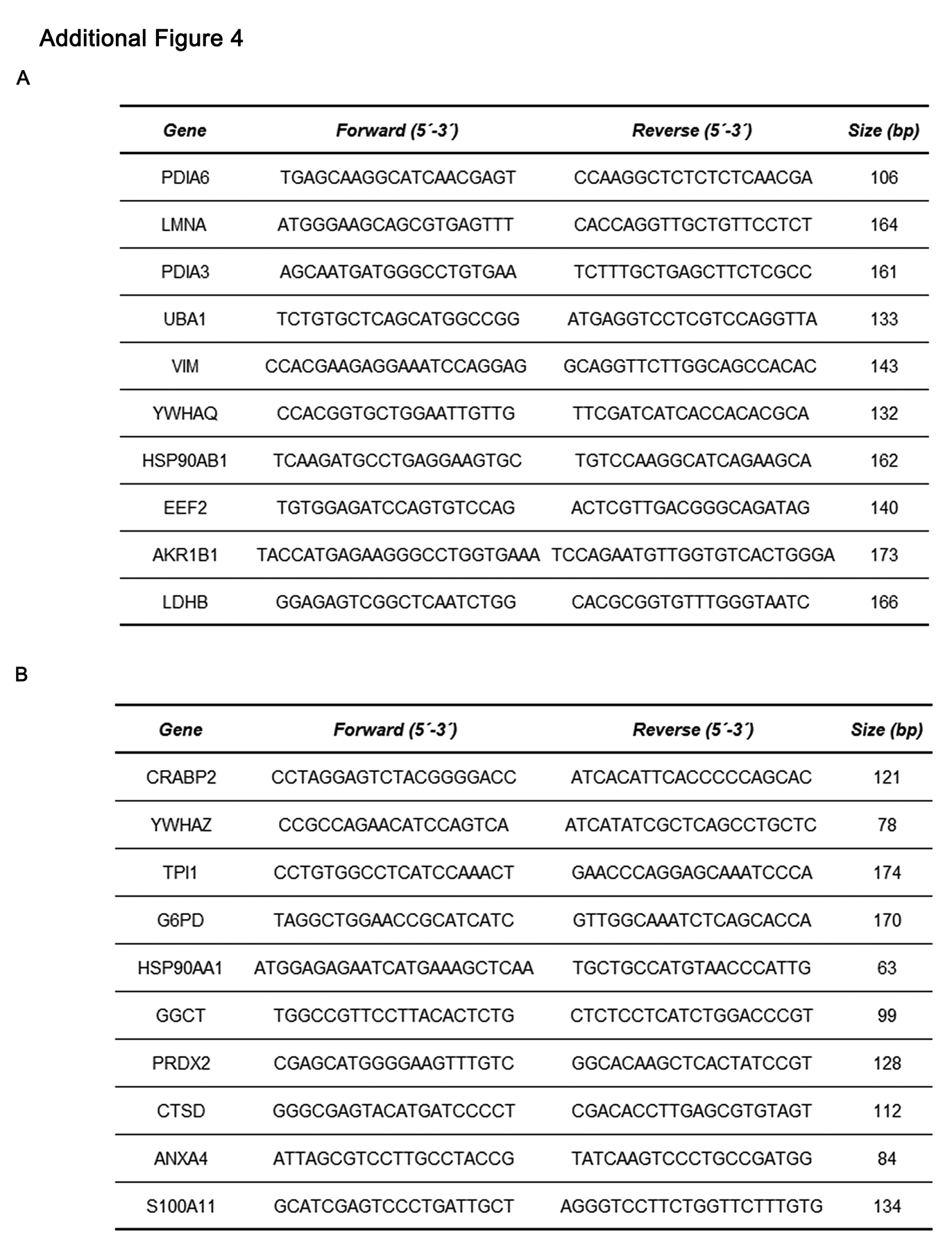

Supplement: Supplementary file 4 — Figure S4. Specific primers for the top-10 upregulated and downregulated molecules among the 50 identified. List of the primers used for the evaluation of the expression levels of the mRNAs that code for the 10 most (A) upregulated and (B) downregulated proteins identified with differential expression levels between MCF7Ecadvar and MCF7pcDNA3 cells by 2D-DIGE and MS. The sequence of the forward and reverse primers, as well as the size of each amplified product, are indicated. (TIF 2455 kb) [file 40170_2019_196_MOESM4_ESM.tif]

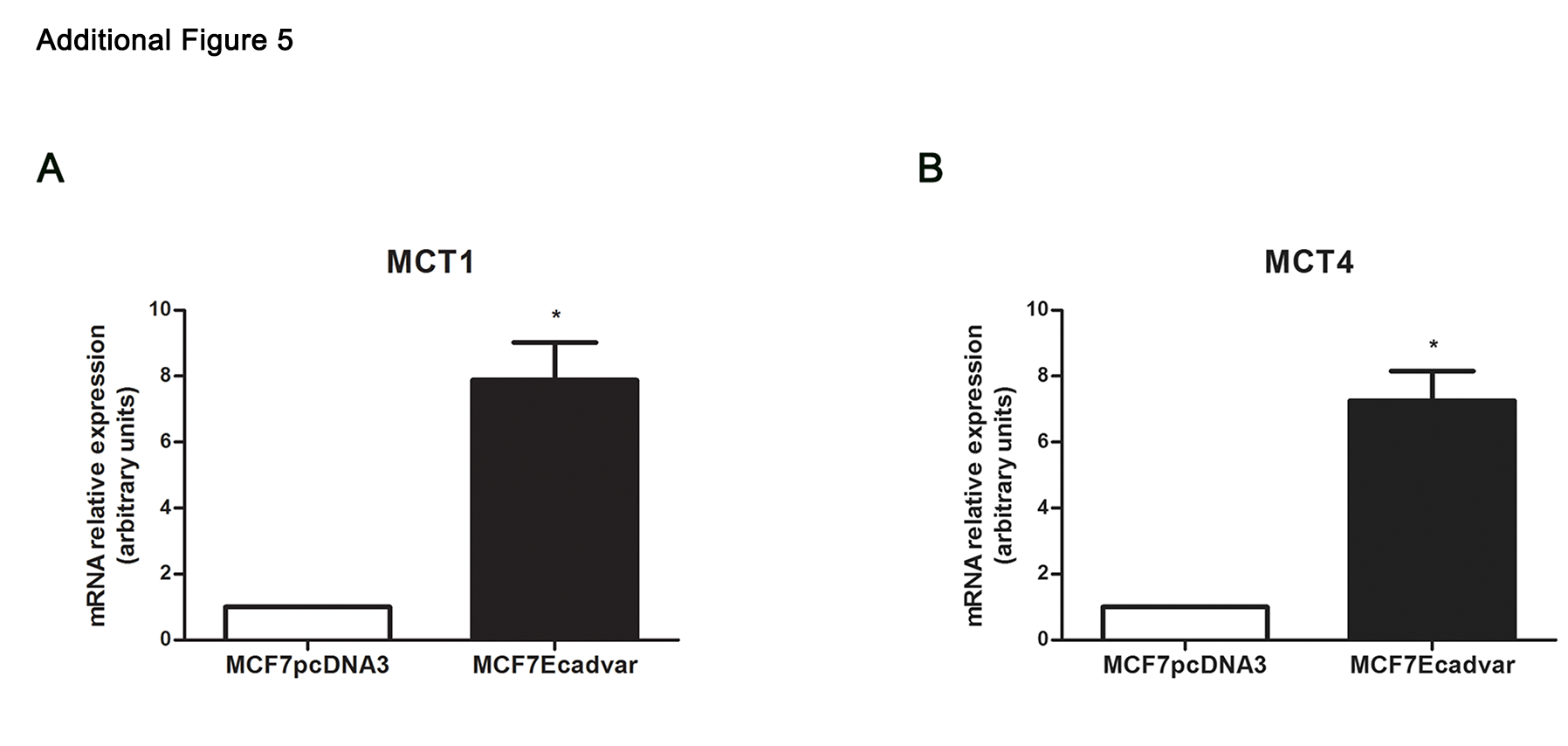

Supplement: Supplementary file 5 — Figure S5. Transcripts expression analysis of MCT1 and MCT4 in MCF7Ecadvar and MCF7pcDNA3 cells. Quantitative expression analysis of (A) MCT1 and (B) MCT4 lactate transporters by real time PCR in MCF7pcDNA3 and MCF7Ecadvar cells. The relative expression was calculated as described in the Materials and Methods section, using GAPDH as the endogenous gene and the MCF7pcDNA3 cell line as reference. *p < 0.05. (TIF 4993 kb) [file 40170_2019_196_MOESM5_ESM.tif]
