# Supplementary material for: Characterization of the molecular changes associated with the overexpression of a novel epithelial cadherin splice variant mRNA in a breast cancer model using proteomics and bioinformatics approaches: identification of changes in cell metabolism and an increased expression of lactate dehydrogenase B
Source: Cancer Metab. 2019 May 9;7:5. doi: 10.1186/s40170-019-0196-9 (PMC6507066; doi:10.1186/s40170-019-0196-9)
Supplement: Supplementary file 3 — Figure S3. Genes associated with human BC. Results obtained with DisGeNET. List of the 5261 human genes that emerged from the 36 terms (“Breast Carcinoma”, “Female Breast Carcinoma”, “Stage 0 Breast Carcinoma”, “Stage IIIA Breast Carcinoma”, “Stage IIIB Breast Carcinoma”, “Invasive Ductal Breast Carcinoma”, “Invasive Lobular Breast Carcinoma”, “Secretory Breast Carcinoma”, “Inflammatory Breast Carcinoma”, “Adenoid Cystic Breast Carcinoma”, “Apocrine Breast Carcinoma”, “Invasive Apocrine Breast Carcinoma”, “Intermediate Grade Ductal Breast Carcinoma In Situ”, “Breast Carcinoma Metastatic in the Skin”, “Breast Cancer 3”, “Breast Cancer Stage II”, “Stage III Breast Cancer AJCC v6”, “Breast Cancer Recurrent”, “Bilateral Breast Cancer”, “Breast Cancer and Pregnancy”, “Breast Cancer, Familial”, “Breast Cancer (non-specific) Premenopausal”, “Contralateral Breast Cancer”, “Unilateral Breast Neoplasms”, “Malignant Neoplasm of Breast”, “Malignant Neoplasm of Female Breast”, “Malignant Neoplasm of Breast Stage I”, “Malignant Neoplasm of Breast Staging”, “Secondary Malignant Neoplasm of Female Breast”, “Triple Negative Breast Neoplasms”, “Mammary Carcinoma, Human”, “Mammary Ductal Carcinoma”, “Mammary Neoplasms”, “Mammary Neoplasms, Human”, “Mammary Neoplasms, Experimental” and “Mammary Tumorigenesis”) found in DisGeNET containing the words “Breast” or “Mammary”, and “Carcinoma”, “Cancer”, “Neoplasms” or “Tumorigenesis”. The 39 genes in common with those that code for the 50 proteins identified by 2D-DIGE and MS as differentially expressed between the MCF7Ecadvar and MCF7pcDNA3 cell lines are highlighted. (PDF 168 kb) [file 40170_2019_196_MOESM3_ESM.pdf]

Additional Figure 3

A2M	ADAM17	AKAP3	ANKRD6	ARHGEF11	ATP6AP2
A2ML1	ADAM22	AKAP4	ANKRD62P1-PARP4P3	ARHGEF12	ATP6V0A2
AAA1	ADAM23	AKAP9	ANO1	ARHGEF2	ATP6V0C
AAAS	ADAM28	AKIP1	ANO7	ARHGEF25	ATP6V1C1
AADAC	ADAM29	AKR1A1	ANP32A	ARHGEF28	ATP7A
AANAT	ADAM33	**AKR1B1**	ANP32B	ARHGEF5	ATP7B
AARS	ADAM8	AKR1B10	ANPEP	ARHGEF7	ATPIF1
AATF	ADAM9	AKR1C1	ANTXR1	ARID1A	ATR
ABCA1	ADAMTS1	AKR1C2	ANTXR2	ARID3B	ATRIP
ABCA4	ADAMTS12	AKR1C3	ANXA1	ARID4A	ATRX
ABCB1	ADAMTS13	AKR1C4	ANXA2	ARID4B	ATXN1
ABCB11	ADAMTS15	AKT1	ANXA3	ARL11	ATXN3L
ABCB5	ADAMTS17	AKT1S1	**ANXA4**	ARL2	AURKA
ABCB6	ADAMTS18	AKT2	ANXA5	ARMC8	AURKB
ABCC1	ADAMTS2	AKT3	ANXA6	ARMC9	AURKC
ABCC10	ADAMTS8	ALB	ANXA7	ARNT	AVP
ABCC11	ADAR	ALCAM	ANXA8	ARNT2	AXIN1
ABCC12	ADCY5	ALDH1A1	AOC1	ARNTL	AXIN2
ABCC2	ADGRF5	ALDH1A2	AP1S2	ARNTL2	AXL
ABCC3	ADGRL2	ALDH1A3	AP2A1	ARPIN	AZGP1
ABCC4	ADH1B	ALDH1B1	APBB1	ARPP21	AZIN2
ABCC5	ADH1C	ALDH1L1	APBB3	ARR3	AZU1
ABCC8	ADH5	ALDH2	APC	ARRB1	B2M
ABCD2	ADH7	ALDH3A1	APC2	ARRB2	B3GALNT2
ABCG1	ADIPOQ	ALDH7A1	APCS	ARRDC3	B3GAT1
ABCG2	ADIPOR1	**ALDOA**	**APEH**	ARSA	B3GNT5
ABI1	ADIPOR2	ALG1	APEX1	ARSF	B4GALNT1
ABL2	ADK	ALK	API5	ARTN	B4GALT7
ABO	ADM	ALKBH5	APOA1	ASAH1	B4GAT1

ACACA	ADO	ALOX12	APOA4	ASAP1	BAAT
ACAD10	ADORA1	ALOX12B	APOB	ASAP3	BABAM1
ACAD8	ADORA2B	ALOX15	APOBEC1	ASCC1	BACE2
ACADSB	ADORA3	ALOX15B	APOBEC3A	ASCL1	BACH1
ACAT1	ADPRHL2	ALOX5	APOBEC3B	ASF1A	BAD
ACD	ADRA1A	ALOX5AP	APOC1	ASF1B	BAG1
ACE	ADRA1D	ALPI	APOC3	ASH2L	BAG3
ACE2	ADRA2B	ALS2CR12	APOD	ASNS	BAGE
ACHE	ADRB1	ALX1	APOE	ASPM	BAK1
ACKR1	ADRB2	AMACR	APP	ASRGL1	BANF1
ACKR2	ADRB3	AMBP	APPBP2	ASS1	BANP
ACKR3	ADRM1	AMD1	APPL1	ASXL1	BAP1
ACKR4	ADSL	AMELX	APPL2	ATAD2	BARD1
ACOT13	AFAP1	AMFR	**APRT**	ATAD3A	BARX2
ACOX2	AFDN	AMH	AQP1	ATAD3B	BASP1
ACP1	AFF3	AMHR2	AQP3	ATAD5	BAX
ACP5	AFP	AMOT	AQP5	ATAT1	BBC3
ACPP	AGER	AMOTL1	AR	ATF1	BBS4
ACSBG1	AGFG1	AMPD1	ARAF	ATF2	BCAP31
ACSL1	AGK	AMPH	ARAP1	ATF3	BCAR1
ACSL4	AGR2	ANAPC4	ARAP3	ATF4	BCAR3
ACSS2	AGR3	ANAPC7	AREG	ATF5	BCAR4
**ACTA2**	AGT	ANG	ARF1	ATF6	BCAS1
**ACTB**	AGTR1	ANGPT1	ARF3	ATG12	BCAS2
ACTG1	AGTR2	ANGPT2	ARF4	ATG4A	BCAS3
ACTN1	AHCY	ANGPTL1	ARF6	ATG7	BCAS4
ACTN4	AHR	ANGPTL2	ARFGEF1	ATIC	BCCIP
ACTR2	AHRR	ANGPTL4	ARFGEF3	ATL1	BCHE
ACVR1	AHSA1	ANGPTL7	ARG1	ATM	BCL10
ACVR1B	AHSG	ANIB1	ARG2	ATN1	BCL2
ACVR2A	BCL6	C3	CASD1	CCNA2	CD63
ACVRL1	BRAP	C3orf35	CASK	CCNB1	CD68

AD11	BRCA1	C4A	CASP1	CCNB2	CD69
AD12	BRCA1P1	C4B	CASP10	CCNC	CD70
AD5	BRCA2	C4B_2	CASP2	CCND1	CD74
ADA	BRCA3	C4BPA	CASP3	CCND2	CD79A
ADAM10	BRCATA	C6orf106	CASP4	CCND3	CD80
ADAM11	BRCC3	C7orf49	CASP5	CCNDBP1	CD82
ADAM12	BRD2	C8orf4	CASP7	CCNE1	CD83
ADAM15	BRD3	CA1	CASP8	CCNE2	CD86
AICDA	BRD4	CA10	CASP9	CCNF	CD8A
AIF1	BRD7	CA11	CASR	CCNG1	CD9
AIFM1	BRD8	CA12	CAST	CCNG2	CD93
AIM2	BRE	CA13	CASZ1	CCNH	CD99
AIMP2	BRF2	CA2	CAT	CCNJ	CDA
AJAP1	BRI3	CA9	CAV1	CCNL2	CDC123
AJUBA	BRI3BP	CABIN1	CAV2	CCNO	CDC14A
AKAP10	BRINP1	CACNA1G	CAV3	CCR2	CDC16
AKAP12	BRIP1	CACNA1H	CBFA2T2	CCR4	CDC20
AKAP13	BRMS1	CACNA1I	CBFA2T3	CCR5	CDC25A
ANIB3	BRMS1L	CACNA2D2	CBFB	CCR6	CDC25B
ANK1	BRS3	CACNA2D3	CBLC	CCR7	CDC25C
ANKHD1	BRSK1	CACNB1	CBR1	CCR9	CDC27
ANKHD1-EIF4EBP3	BRWD3	CACUL1	CBR3	CCRL2	CDC34
ANKLE1	BSG	CACYBP	CBS	CCT2	CDC42
ANKRD11	BST2	CAD	CBX1	CCT4	CDC42BPA
ANKRD12	BTC	CADM1	CBX3	CCT5	CDC42BPB
ANKRD30A	BTD	CALB1	CBX4	CD109	CDC6
ANKRD36B	BTF3P11	CALCA	CBX5	CD14	CDC7
ANKRD46	BTG1	CALCOCO1	CBX7	CD151	CDCA8
ARGLU1	BTG2	CALCR	CBX8	CD160	CDCP1
ARHGAP1	BTK	CALD1	CCAR1	CD163	CDH1
ARHGAP10	BTLA	CALM1	CCAR2	CD177	CDH11

ARHGAP11A	BTRC	CALM2	CCAT2	CD1A	CDH13
ARHGAP24	BUB1	CALM3	CCDC170	CD1D	CDH2
ARHGAP31	BUB1B	CALR	CCDC6	CD200	CDH23
ARHGAP32	BUB3	CAMK1	CCDC8	CD200R1	CDH3
ARHGDIA	BUD31	CAMK1D	CCDC88A	CD24	CDH5
ARHGDIG	BYSL	CAMK2B	CCDC88C	CD27	CDK1
ARHGEF1	C10orf10	CAMK2G	CCK	CD274	CDK10
ATP11A	C10orf11	CAMKMT	CCL1	CD276	CDK11A
ATP1B1	C10orf88	CAMLG	CCL14	CD28	CDK11B
ATP1B2	C10orf90	CAMP	CCL16	CD33	CDK12
ATP2B1	C14orf166	CANX	CCL18	CD34	CDK19
ATP2B2	C16orf82	CAP1	CCL19	CD36	CDK2
ATP2B3	C17orf97	CAPG	CCL2	CD38	CDK3
ATP2B4	C18orf8	CAPN2	CCL20	CD3D	CDK4
ATP2C1	C19orf48	CAPNS1	CCL21	CD4	CDK5
ATP2C2	C1GALT1	CAPZB	CCL22	CD40	CDK5R1
ATP6AP1	C1orf109	CARD10	CCL25	CD40LG	CDK6
BCL2A1	C1orf52	CARD14	CCL27	CD44	CDK7
BCL2L1	C1orf64	CARD16	CCL28	CD46	CDK8
BCL2L10	C1QA	CARD8	CCL3	CD47	CDK9
BCL2L11	C1QBP	CARM1	CCL4	CD48	CDKL2
BCL2L12	C1QL1	CASC1	CCL4L1	CD53	CDKN1A
BCL2L14	C20orf181	CASC16	CCL4L2	CD55	CDKN1B
BCL2L2	C21orf33	CASC22	CCL5	CD59	CDKN1C
BCL2L2-PABPN1	C22orf29	CASC3	CCL7	CD5L	CDKN2A
BCL3	C2orf40	CASC8	CCNA1	CD6	CDKN2B
CHEK1	CLU	CPNE3	CSNK2B	CXCL2	DAP3
CHEK2	CLUL1	CPOX	CSPG4	CXCL3	DAPK1
CHFR	CMA1	CPSF7	CSPP1	CXCL5	DAPK2
CHGB	CMC2	CPT1A	CSRP2	CXCL8	DAPK3
CHI3L1	CMD1B	CPT2	CST2	CXCL9	DAZ1

CHIT1	CMM	CPVL	CST3	CXCR1	DBA2
CHKA	CMPK1	CR1	CST5	CXCR2	DBH-AS1
CHM	CMPK2	CRABP1	CST6	CXCR3	DBI
CHN2	CMTR1	**CRABP2**	CST9	CXCR4	DBP
CHPT1	CNBP	CRAT	CSTA	CXCR5	DCAF1
CHRNA4	CNKSR1	CRCP	CT45A1	CXCR6	DCAF7
CHRNA9	CNN3	CREB1	CT55	CXXC5	DCC
CHST11	CNNM3	CREB3	CT83	CYB5A	DCD
CHST3	CNOT7	CREB3L1	CTAA1	CYB5R3	DCK
CHUK	CNOT8	CREB3L4	CTAG1A	CYBA	DCLRE1C
CIB1	CNOT9	CREBBP	CTAG1B	CYBB	DCN
CIB2	CNR1	CREM	CTBP1	CYFIP2	DCPS
CIC	CNR2	CRH	CTBP2	CYGB	DCTN1
CIITA	CNTLN	CRHR1	CTCF	CYLD	DCTN3
CIRBP	CNTN3	CRHR2	CTCFL	CYP11A1	DCTN4
CISD1	CNTN6	CRIP1	CTDSP1	CYP11B1	DCTN5
CISD2	CNTNAP1	CRIP2	CTDSPL	CYP11B2	DCTN6
CISH	CNTNAP4	CRIPAK	CTGF	CYP17A1	DDB2
CITED1	CNTRL	CRISP2	CTHRC1	CYP19A1	DDHD2
CITED2	CNTROB	CRISPLD2	CTLA4	CYP1A1	DDIAS
CITED4	COASY	CRK	CTNNB1	CYP1A2	DDIT3
CIZ1	COIL	CRKL	CTNNBIP1	CYP1B1	DDIT4
CKAP2	COL11A1	CRLF3	CTNND1	CYP24A1	DDR1
CKAP4	COL11A2	CRLS1	CTNND2	CYP26A1	DDR2
CKAP5	COL12A1	CRMP1	CTR9	CYP27B1	DDT
CKB	COL15A1	CRP	CTRL	CYP2A6	DDX1
CKS1B	COL18A1	CRTC1	CTSB	CYP2B6	DDX10
CKS1BP7	COL1A1	CRTC2	CTSC	CYP2C18	DDX17
CLASP1	COL1A2	CRX	**CTSD**	CYP2C19	DDX18
CLCA1	COL4A1	CRY1	CTSH	CYP2C8	DDX19A
CLCA2	COL4A2	CRY2	CTSK	CYP2C9	DDX20
CLCA4	COL4A3BP	CRYAB	CTSL	CYP2D6	DDX21

CLCN2	COL7A1	CRYGC	CTSO	CYP2E1	DDX3X
CLDN1	COMMD3-BMI1	CRYGEP	CTSZ	CYP3A4	DDX43
CLDN16	COMP	CRYZ	CTTN	CYP3A43	DDX46
CLDN2	COMT	CSE1L	CTU1	CYP3A5	DDX5
CLDN3	COPS5	CSF1	CUEDC2	CYP3A7	DDX53
CLDN4	COPS6	CSF1R	CUL1	CYP3A7-CYP3A51P	DDX54
CLDN5	COPS8	CSF2	CUL2	CYP4B1	DEC1
CLDN6	CORO1A	CSF3	CUL3	CYP4F3	DECR1
CLDN7	CORO1C	CSGALNACT1	CUL4A	CYP4Z1	DEF6
CLDND1	CORT	CSH1	CUL5	CYP4Z2P	DEFA1
CLEC10A	COTL1	CSH2	CUX1	CYR61	DEFA1B
CLEC16A	COX1	CSHL1	CWF19L2	CYSLTR1	DEFB4A
**CLIC1**	COX11	CSK	CX3CL1	CYSLTR2	DEFB4B
CLIC3	COX2	CSMD1	CX3CR1	CYTB	DEK
CLIC4	COX4I1	CSN1S1	CXADR	DAAM1	DENR
CLIP1	COX5A	CSN2	CXADRP1	DAB2	DEPTOR
CLIP2	COX8A	CSN3	CXCL1	DAB2IP	DERL1
CLK2	CP	CSNK1A1	CXCL10	DACH1	DES
CLN3	CPA1	CSNK1D	CXCL12	DACT1	DFFA
CLOCK	CPB1	CSNK1E	CXCL13	DAD1	DFNA5
CLPTM1L	CPD	CSNK2A1	CXCL14	DAG1	DGCR8
CLSPN	CPEB2	CSNK2A2	CXCL16	DAP	DGKA
DOK7	EDIL3	ELK4	ERBB2	F2R	FBXL17
DOPEY1	EDN1	ELL3	ERBB3	F2RL1	FBXL7
DOT1L	EDN2	ELMO1	ERBB4	F2RL2	FBXO10
DPEP1	EDN3	ELN	ERCC1	F2RL3	FBXO11
DPF3	EDNRA	ELOB	ERCC2	F3	FBXO18
DPP4	EDNRB	ELOC	ERCC3	F5	FBXO28
DPP8	EED	ELOF1	ERCC4	F7	FBXO31
DPT	EEF1A1	ELOVL1	ERCC5	F8	FBXO32
DPYD	EEF1A2	ELP3	ERCC6	F9	FBXO6

DPYSL2	EEF1B2	EMB	ERCC8	FA2H	FBXW4
DRAM1	EEF1B2P2	EMC1	EREG	FAAH	FBXW7
DRD2	EEF1D	EMD	ERG	FABP3	FCER1A
DRD3	**EEF2**	EME1	ERLIN2	FABP4	FCGR1B
DRG1	EEF2K	EMG1	ERN1	FABP5	FCGR2A
DROSHA	EFEMP1	EMILIN2	**ERP29**	FABP7	FCGR2B
DSC2	EFEMP2	EMP1	ERRFI1	FABP9	FCGR2C
DSC3	EFNA1	EMSY	ERV3-1	FADD	FCGR3A
DSCAM-AS1	EFNA2	EN2	ERVK-11	FADS2	FCGR3B
DSN1	EFNA3	ENAH	ERVK-12	FAF1	FCRL3
DSP	EFNA5	ENC1	ERVK-15	FAH	FECH
DST	EFNB2	ENDOG	ERVK-18	FAM102A	FEN1
DTL	EGF	ENDOU	ERVK-19	FAM107B	FERMT1
DTX3	EGFL7	ENG	ERVK-2	FAM124B	FERMT2
DUOXA1	EGFR	ENGASE	ERVK-20	FAM175A	FERMT3
DUSP1	EGLN1	ENO1	ERVK-22	FAM19A4	FEV
DUSP2	EGLN2	ENO2	ERVK-6	FAM215A	FEZ1
DUSP22	EGLN3	ENOSF1	ERVK-7	FAM32A	FFAR1
DUSP23	EGR1	ENOX2	ERVK-9	FAM46A	FGA
DUSP4	EGR3	ENPEP	ERVW-1	FAM58A	FGD1
DUSP5	EHF	ENPP1	ERVW-4	FAM64A	FGD3
DUSP6	EHMT1	ENPP2	ESPL1	FAM83A	FGD5
DVL1	EHMT2	ENTPD5	ESR1	FAM83B	FGF1
DVL1P1	EI24	EP300	ESR2	FAM83D	FGF10
DVL2	EIF2AK2	EPAS1	ESRP1	FAN1	FGF13
DYM	EIF2AK3	EPB41L3	ESRRA	FANCA	FGF17
DYNC2H1	EIF2S2	EPB41L4B	ESRRB	FANCB	FGF18
DYNLL1	EIF3A	EPB41L5	ESRRG	FANCC	FGF19
DYRK2	EIF3E	EPCAM	ESYT1	FANCD2	FGF2
DYX1C1	EIF3H	EPG5	ETF1	FANCE	FGF3
DZIP1	EIF4A1	EPHA1	ETS1	FANCF	FGF4
E2F1	EIF4A2	EPHA10	ETS2	FANCG	FGF7

E2F2	EIF4B	EPHA2	ETV1	FANCI	FGF8
E2F3	EIF4E	EPHA3	ETV3	FANCL	FGF9
E2F4	EIF4E3	EPHA4	ETV4	FANCM	FGFBP1
E2F5	EIF4EBP1	EPHA5	ETV5	FAP	FGFR1
E2F6	EIF4G1	EPHA7	ETV6	FAS	FGFR2
E2F7	EIF4G2	EPHA8	ETV7	FASLG	FGFR3
EBAG9	EIF5A	EPHB2	EVL	FASN	FGFR4
EBF1	EIF6	EPHB4	EXO1	FASTKD2	FGFRL1
EBI3	ELAC2	EPHB6	EXOSC6	FAT1	FGR
EBP	ELANE	EPHX1	EXT1	FAT4	FHIT
ECD	ELAVL1	EPM2AIP1	EXTL3	FATE1	FHL1
ECE1	ELAVL2	EPO	EYA1	FBL	FHL2
ECHDC1	ELF1	EPOR	EYA2	FBLIM1	FHL3
ECHS1	ELF3	EPS15	EZH2	FBLN1	FIBP
ECM1	ELF4	EPS8	EZR	FBLN2	FKBP1A
EDA	ELF5	EPSTI1	F10	FBLN5	FKBP1AP1
EDA2R	ELK1	EPX	F11R	FBN2	FKBP1AP2
EDAR	ELK3	ERAL1	F2	**FBP1**	FKBP1AP3
FRZB	GATA5	GLI2	GRB7	HAVCR1	HIST1H4H
FSCN1	GATA6	GLI3	GREB1	HBA1	HIST1H4I
FSD1	GATAD2B	GLO1	GREM1	HBB	HIST1H4J
FSD1L	GATM	GLRX	GRHL2	HBEGF	HIST1H4K
FSHB	GBP1	GLRX3	GRIN1	HBP1	HIST1H4L
FSHR	GC	GLS	GRIP1	HCA1	HIST2H4A
FSIP1	GCG	GLS2	GRK2	HCAR2	HIST2H4B
FST	GCLC	GLTSCR2	GRK3	HCAR3	HIST4H4
FSTL3	GCNT2	GLYAT	GRK4	HCC	HIVEP1
FTH1	GCY	GLYCAM1	GRM1	HCCAT5	HIVEP2
FTL	GDA	GM2A	GRN	HCLS1	HIVEP3
FTO	GDE1	GMNN	GRP	HCN1	HJURP
FURIN	GDF1	GMPR2	GRPR	HDAC1	HK1
FUS	GDF10	GNA12	GSDMB	HDAC2	HK2

FUT1	GDF15	GNA13	GSK3B	HDAC3	HLA-A
FUT2	GDF2	GNAI2	GSN	HDAC4	HLA-B
FUT3	GDF3	GNAO1	GSR	HDAC6	HLA-C
FUT4	GDF5	GNAS	GSS	HDAC7	HLA-DOA
FXYD3	GDF9	GNB1	GSTA1	HDAC8	HLA-DOB
FXYD5	GDNF	GNB3	GSTA2	HDAC9	HLA-DPB1
FYN	GDPD5	GNG12-AS1	GSTK1	HDC	HLA-DQA1
FZD1	GEMIN2	GNL3	GSTM1	HDDC3	HLA-DQB1
FZD2	GEMIN4	GNMT	GSTM2	HDLBP	HLA-DRB1
FZD5	GEN1	GNRH1	GSTM3	HEATR1	HLA-G
FZD7	GFAP	GNRHR	GSTM4	HEATR6	HM13
FZD8	GFM1	GOLGA2	GSTM5	HECW1	HMBS
FZR1	GFPT1	GOLPH3	GSTO1	HELLS	HMGA1
G0S2	GFRA1	GOLT1A	GSTO2	HEMC	HMGA2
G3BP1	GFRA3	GOPC	GSTP1	HEPACAM	HMGB1
G3BP2	**GGCT**	GORASP1	GSTT1	HES1	HMGB2
**G6PD**	GGH	GOSR1	GSTZ1	HES6	HMGB3
GAA	GGN	GOT2	GTF2A1	HEXIM1	HMGCR
GAB1	GGT1	GP1BA	GTF2E1	HEY	HMGCS2
GAB2	GGTA1P	GPAA1	GTF2F1	HEY1	HMGN1
GABARAP	GH1	GPAT2	GTF2H1	HEY2	HMGN5
GABARAPL1	GHR	GPAT3	GTF3A	HEYL	HMMR
GABPA	GHRH	GPATCH2	GTPBP4	HFE	HMOX1
GABRA3	GHRHR	GPC1	GTSE1	HFM1	HNF4A
GABRP	GHRL	GPC2	GUCY1A2	HGF	HNMT
GADD45A	GHSR	GPC3	GUSB	HGS	HNRNPA1
GADD45G	GIGYF1	GPC6	GZMB	HHAT	HNRNPA2B1
GADD45GIP1	GIGYF2	GPD1	GZMM	HHEX	HNRNPAB
GAL	GINS2	GPER1	H19	HHLA2	HNRNPC
GALNS	GIPC1	**GPI**	H1F0	HIC1	HNRNPD
GALNT12	GIPC2	GPNMB	H2AFJ	HIF1A	HNRNPDL
GALNT14	GIPC3	GPR17	H2AFX	HIF1AN	**HNRNPH1**

GALNT4	GIT1	GPR182	H2AFY	HIP1	HNRNPK
GALNT6	GJA1	GPR42	H2AFZ	HIPK2	HNRNPL
GAPDH	GJA3	GPRC5A	H3F3AP6	HIST1H1C	HNRNPR
GARS	GJA8	GPSM2	H3F3B	HIST1H2BC	HNRNPU
GAS1	GJB1	GPX1	HACD1	HIST1H2BE	HORMAD1
GAS1RR	GJB2	GPX2	HADHA	HIST1H2BK	HOTAIR
GAS5	GJB6	GPX3	HADHB	HIST1H2BM	HOXA@
GAS6	GJC2	GPX4	HAGH	HIST1H4A	HOXA1
GAST	GKN1	GPX6	HAMP	HIST1H4B	HOXA10
GATA1	GLB1	GPX7	HARS	HIST1H4C	HOXA5
GATA2	GLCE	GRAP2	HAS1	HIST1H4D	HOXA9
GATA3	GLG1	GRB14	HAS2	HIST1H4E	HOXB@
GATA4	GLI1	**GRB2**	HAT1	HIST1H4F	HOXB1
HSPB3	IGF2BP3	IL3	ISYNA1	KCNH4	KLF5
HSPB8	IGF2R	IL31RA	ITCH	KCNH8	KLF6
HSPBP1	IGFALS	IL32	ITFG1	KCNIP3	KLF8
HSPD1	IGFBP1	IL33	ITGA2	KCNJ3	KLHL1
HSPG2	IGFBP2	IL4	ITGA2B	KCNK5	KLK1
HSPH1	IGFBP3	IL4R	ITGA3	KCNK9	KLK10
HSR	IGFBP4	IL5	ITGA4	KCNMA1	KLK11
HTATIP2	IGFBP5	IL6	ITGA5	KCNN3	KLK12
HTC2	IGFBP6	IL6R	ITGA6	KCNQ1OT1	KLK13
HTN3	IGFBP7	IL6ST	ITGA9	KDM1A	KLK14
HTR2A	IGFBPL1	IL7	ITGAL	KDM1B	KLK15
HTR3A	IGHG3	IL7R	ITGAM	KDM2A	KLK2
HTR3B	IGHMBP2	ILF3	ITGAV	KDM2B	KLK3
HTR3C	IGK	ILK	ITGAX	KDM3A	KLK4
HTR3D	IGKC	IMMP1L	ITGB1	KDM3B	KLK5
HTR3E	IGSF1	IMMP2L	ITGB2	KDM4A	KLK6
HTRA1	IHH	IMMT	ITGB3	KDM4B	KLK7
HTT	IKBKB	IMP3	ITGB3BP	KDM4C	KLK8
HUNK	IKBKE	IMPDH1	ITGB4	KDM5A	KLK9

HUS1	IKBKG	INCENP	ITGB5	KDM5B	KLKB1
HVCN1	IKZF2	ING1	ITGB6	KDM5C	KLLN
HYAL1	IKZF3	ING2	ITGBL1	KDM6A	KLRC4-KLRK1
HYAL2	IL10	ING4	ITIH1	KDR	KLRK1
IAPP	IL11	INHA	ITIH2	KEAP1	KMT2A
IARS	IL11RA	INHBA	ITIH4	KHDRBS1	KMT2B
IBSP	IL12A	INHBB	ITIH5	KHDRBS3	KMT2C
ICAM1	IL12B	INPP4B	ITK	KHSRP	KMT2D
ICAM4	IL12RB2	INPP5J	ITM2B	KIAA0100	KMT5A
ICAM5	IL13	INPPL1	ITPR1	KIAA1524	KMT5C
ICK	IL13RA2	INS	ITSN2	KIF11	KNG1
ICOS	IL15	INSIG1	JADRR	KIF12	KPNA2
ID1	IL15RA	INSIG2	JAG1	KIF14	KPNB1
ID3	IL16	INSL4	JAG2	KIF18A	KRAS
ID4	IL17A	INSR	JAK1	KIF20A	KRIT1
IDH1	IL17B	INTS1	JAK2	KIF20B	KRT13
IDH2	IL17C	INTS2	JAK3	KIF22	KRT14
IDO1	IL17D	INTS6	JMJD1C	KIF23	KRT15
IDUA	IL17F	INTU	JMJD6	KIF24	KRT17
IER3	IL17RB	IPO13	JMY	KIF26B	KRT18
IFI16	IL18	IQGAP1	JTB	KIF2A	KRT19
IFI27	IL18R1	IQSEC1	JUN	KIF2C	KRT2
IFI30	IL19	IRAIN	JUNB	KIF3C	KRT20
IFIT3	IL1A	IRAK1	JUND	KIF5A	KRT5
IFITM1	IL1B	IRAK3	JUP	KIFC1	KRT6A
IFITM10	IL1R1	IRF1	KANK1	KIFC3	KRT7
IFNA1	IL1RN	IRF4	KANK2	KIN	KRT71
IFNA13	IL2	IRF5	KAT2A	KIR2DL5B	KRT8
IFNB1	IL20	IRF6	KAT2B	KIR2DS2	KRT80
IFNG	IL20RA	IRF7	KAT5	KISS1	KRT88P
IFNGR1	IL21	IRF8	KAT6A	KISS1R	KSR1
IFNGR2	IL21R	IRF9	KAT7	KIT	L1CAM

IFT122	IL22	IRS1	KAT8	KITLG	L3MBTL1
IGBP1	IL23R	IRS2	KATNA1	KL	L3MBTL4
IGDCC3	IL24	IRX2	KATNB1	KLB	LACRT
IGF1	IL25	IRX5	KCNA1	KLF10	LAG3
IGF1R	IL27	ISG15	KCNA3	KLF15	LALBA
IGF2	IL2RA	ISG20	KCNA5	KLF17	LAMA3
IGF2BP1	IL2RB	ISL1	KCNH1	KLF2	LAMA5
IGF2BP2	IL2RG	IST1	KCNH2	KLF4	LAMB1
LIN9	LRPAP1	MAOA	MC4R	MHS6	MIR15A
LINC00160	LRPPRC	MAP1LC3A	MCAM	MIA	MIR15B
LINC00273	LRRC15	MAP1LC3B	MCAT	MIA3	MIR17
LINC00328	LRRC26	MAP1S	MCC	MIB1	MIR17HG
LINC00472	LRRC4	MAP2	MCF2L	MIB2	MIR181C
LINC00914	LRRC49	MAP2K1	MCL1	MICA	MIR182
LINC01016	LRRC59	MAP2K3	MCM2	MICB	MIR183
LINC01193	LRRK2	MAP2K4	MCM3	MICE	MIR184
LINC01194	LRTOMT	MAP2K5	MCM3AP	MICU1	MIR185
LINC02210-CRHR1	LRWD1	MAP2K7	MCM4	MIEN1	MIR187
LINC-PINT	LSAMP	MAP3K1	MCM5	MIER1	MIR18A
LINC-ROR	LSM1	MAP3K10	MCM6	MIER3	MIR18B
LIPF	LSM2	MAP3K11	MCM7	MIF	MIR190A
LIPG	LSP1	MAP3K12	MCPH1	MIF-AS1	MIR190B
LITAF	LSR	MAP3K14	MCS	MIIP	MIR191
LLGL1	LSS	MAP3K2	MCTS1	MIP	MIR192
LLGL2	LTA	MAP3K20	MCU	MIR100	MIR193A
LMLN	LTB4R	MAP3K3	MDC1	MIR100HG	MIR193B
**LMNA**	LTB4R2	MAP3K5	MDH2	MIR101-2	MIR195
LMO2	LTBP1	MAP3K7	MDK	MIR106B	MIR196A2
LMO4	LTBP4	MAP3K7CL	MDM2	MIR107	MIR196B
LMTK3	LTBR	MAP3K8	MDM4	MIR10A	MIR197
LNPEP	LTF	MAP3K9	**ME1**	MIR10B	MIR1972-2

LNPK	LUM	MAP4K4	MECOM	MIR122	MIR199A1
LOC100128274	LXN	MAPK1	MECP2	MIR1245A	MIR199A2
LOC100128922	LY6K	MAPK12	MED1	MIR1258	MIR199B
LOC100288966	LYN	MAPK13	MED12	MIR125A	MIR19A
LOC100505909	LYNX1	MAPK14	MED13	MIR125B1	MIR200A
LOC100506248	LYPD4	MAPK15	MED14	MIR125B2	MIR200B
LOC100507703	LYPD5	MAPK3	MED15	MIR126	MIR200C
LOC101930123	LYVE1	MAPK6	MED19	MIR127	MIR202
LOC102723971	LZTS1	MAPK7	MED25	MIR128-1	MIR203A
LOC102724023	LZTS2	MAPK8	MED28	MIR128-2	MIR204
LOC105369230	MACC1	MAPK9	MEF2A	MIR1290	MIR205
LOC107987479	MACROD1	MAPKAPK2	MEF2C	MIR1303	MIR206
LOC400499	MAD1L1	MAPRE1	MEFV	MIR130A	MIR208A
LOC400927-CSNK1E	MAD2L1	MAPT	MEGF8	MIR130B	MIR20A
LOC90784	MAD2L2	MARCH1	MEGF9	MIR132	MIR20B
LONP1	MAF	MARCH8	MEIS1	MIR133A1	MIR21
LOR	MAFD2	MARCKS	MELK	MIR134	MIR210
LOX	MAGEA1	MARCKSL1	MEMO1	MIR135B	MIR211
LOXL1	MAGEA3	MARK2	MEN1	MIR137	MIR212
LOXL2	MAGEC1	MARVELD1	MEPE	MIR139	MIR214
LOXL3	MAGEC2	MAS1	MERTK	MIR140	MIR219A1
LOXL4	MAGED2	MAST2	MEST	MIR141	MIR22
LPA	MAGED4	MAT1A	MET	MIR142	MIR221
LPAR1	MAGED4B	MAT2A	METTL6	MIR143	MIR222
LPAR2	MAGEE1	MATK	METTL8	MIR144	MIR223
LPAR3	MAGEE2	MAVS	MFGE8	MIR145	MIR22HG
LPAR6	MAGEH1	MAZ	MFN2	MIR146A	MIR23A
LPCAT1	MAGI2	MB	MFT2	MIR146B	MIR23B
LPL	MAGI3	MBD2	MGA	MIR147A	MIR24-1
LPP	MAGT1	MBL2	MGAT1	MIR148A	MIR24-2
LPXN	MAK16	MBOAT4	MGAT3	MIR148B	MIR25

LRIG1	MAL	MBP	MGAT5	MIR149	MIR26B
LRP1	MAL2	MBTPS1	MGEA5	MIR150	MIR27A
LRP2	MALAT1	MC1R	MGLL	MIR151A	MIR27B
LRP5	MALT1	MC2R	MGMT	MIR152	MIR28
LRP6	MAML1	MC3R	MGP	MIR155	MIR296
MIR485	MIR96	MRPL13	MTRR	NANOS1	NEK3
MIR486-1	MIR98	MRPL19	MTSS1	NAP1L1	NEK7
MIR487A	MIR99A	MRPL23	MTTP	NAT1	NEK8
MIR489	MIR99B	MRPL28	MTUS1	NAT2	NEK9
MIR491	MIRLET7A2	MRPL36	MTX1	NAV1	NELFB
MIR492	MIRLET7B	MRPL41	MUC1	NBAT1	NELFE
MIR494	MIRLET7C	MRPL9	MUC15	NBN	NELL2
MIR495	MIRLET7E	MRPS22	MUC16	NBR1	NES
MIR496	MIRLET7G	MRPS23	MUC17	NCAM1	NET1
MIR497	MIRLET7I	MRPS28	MUC2	NCAM2	NEU1
MIR498	MKI67	MRPS30	MUC3	NCAPD2	NEURL1
MIR499A	MKL1	MRPS7	MUC3A	NCAPG2	NEUROD1
MIR502	MKNK1	MS	MUC3B	NCBP2	NF1
MIR503	MKNK2	MS4A1	MUC4	NCF2	NF2
MIR505	MKS1	MSC	MUC5AC	NCF4	NFAT5
MIR506	MLC1	MSH2	MUC5B	NCKAP1	NFATC1
MIR510	MLF2	MSH3	MUC6	NCL	NFATC2
MIR526B	MLH1	MSH4	MUCL1	NCOA1	NFATC3
MIR532	MLH3	MSH6	MUL1	NCOA2	NFATC4
MIR542	MLLT11	MSI1	MUS81	NCOA3	NFE2
MIR562	MLN	MSLN	MUSTN1	NCOA4	NFE2L1
MIR568	MLRL	MSMB	MUT	NCOA6	NFE2L2
MIR569	MLXIP	MSN	MUTYH	NCOA7	NFIB
MIR573	MLXIPL	MSR1	MVD	NCOR1	NFIC
MIR574	MME	MSRA	MVP	NCOR2	NFIL3
MIR578	MMP1	MST1	MXD1	NCSTN	NFKB1
MIR584	MMP10	MST1R	MXI1	ND1	NFKB2

MIR590	MMP11	MSTO1	MYB	ND3	NFKBIA
MIR592	MMP12	MSX1	MYBBP1A	ND5	NFKBIE
MIR605	MMP13	MSX2	MYBL2	NDC80	NFYA
MIR608	MMP14	MT1A	MYC	NDE1	NGDN
MIR612	MMP17	MT1B	MYCBPAP	NDN	NGF
MIR621	MMP2	MT1E	MYCL	NDP	NGFR
MIR630	MMP25	MT1F	MYCN	NDRG1	NHS
MIR638	MMP26	MT1G	MYD88	NDRG2	NID1
MIR652	MMP3	MT1H	MYDGF	NDST1	NID2
MIR655	MMP7	MT1IP	MYEOV	NDUFAF3	NIN
MIR660	MMP8	MT1JP	MYH10	NDUFAF4	NINL
MIR661	MMP9	MT1L	MYH11	NDUFB3	NISCH
MIR663A	MMRN1	MT1M	MYH2	NDUFB9	NKD1
MIR671	MNAT1	MT1X	MYH7B	NDUFS3	NKD2
MIR675	MOK	MT2A	MYH9	NDUFS4	NKILA
MIR6861	MORF4	MT3	MYLIP	NDUFS7	NKX3-1
MIR6875	MORF4L1	MTA1	MYLK	NEAT1	NLK
MIR708	MOS	MTA2	MYO10	NECAB3	NLRP1
MIR7-1	MPC1	MTA3	MYOCD	NECTIN1	NLRP2
MIR7-2	MPEG1	MTAP	MYOD1	NECTIN3	NLRP3
MIR7-3	MPG	MTBP	MYOF	NECTIN4	NLRP6
MIR744	MPHOSPH10	MTDH	MYOM2	NEDD4	NM
MIR762	MPL	MTG1	MYT1	NEDD8	NMBR
MIR873	MPO	MTHFD1	MZF1	NEDD9	NME1
MIR874	MPRIP	MTHFD2	NAA10	NEFH	NME1-NME2
MIR9-1	MPST	MTHFR	NAA16	NEFL	**NME2**
MIR920	MPZL1	MTMR3	NAA25	NEFM	NME3
MIR93	MPZL2	MTNR1A	NAB2	NEIL1	NMI
MIR934	MR1	MTNR1B	NAF1	NEIL2	NMU
MIR940	MRC1	MTO1	NAIP	NEK1	NNMT
MIR944	MRC2	MTOR	NAMPT	NEK10	NOC2L
MIR95	MRE11	MTR	NANOG	NEK2	NOD1

NRBF2	OPRM1	PARVB	PECAM1	PIK3R2	PLPP5
NRCAM	OPTN	PAWR	PEG10	PIK3R3	PLPPR5
NRDC	OR10A4	PAX2	PEG3	PIM1	PLS3
NRF1	OR10J3	PAX5	PELP1	PIN1	PLTP
NRG1	ORAI1	PAX6	PEMT	PINK1	PLXNB1
NRG2	ORAI3	PBK	PEPD	PINX1	PMAIP1
NRG3	OSCP1	PBOV1	PER1	PIP	PMEPA1
NRG4	OSGIN1	PBRM1	PER2	PIP4K2B	PML
NRIP1	OSM	PBX1	PER3	PIP5K1A	PMP22
NRP1	OSMR	PBXIP1	PERP	PIPOX	PMS1
NRP2	OTUB1	PC	PES1	PITPNM1	PMS2
NRXN2	OTUD4	PCAP	PEX14	PITX1	PNCK
NSD2	OTX1	PCBP1	PF4	PITX2	PNKD
NSD3	OTX2	PCBP4	PFDN4	PIWIL1	PNO1
NSG1	OXT	PCDH8	PFKFB3	PIWIL2	PNP
NSMCE2	OXTR	PCDHA@	PFKFB4	PIWIL4	PNPLA2
NSMF	P2RX5	PCDHB@	PFKM	PKD1	POC1A
NSUN2	P2RX7	PCDHG@	PFKP	PKD2	POC1B-GALNT4
NSUN5	P2RY2	PCDHGB6	PFN1	PKD2L1	PODXL
NT5C2	P3H1	PCGF2	PFN2	PKIB	POLB
NT5E	P3H2	PCLAF	**PGAM1**	PKLR	POLD1
NTF3	P3H3	PCNA	PGC	PKM	POLD3
NTF4	P4HA1	PCNT	PGF	PKMYT1	POLDIP2
NTHL1	P4HA2	PCP4	PGK1	PKN2	POLE
NTN1	P4HB	PCSK6	**PGLS**	PLA2G10	POLG
NTN4	PA2G4	PCSK7	PGP	PLA2G1B	POLH
NTRK1	PABPC1	PCYT1A	PGPEP1	PLA2G2A	POLI
NTRK3	PABPC1P10	PDC	PGR	PLA2G4A	POLK
NTS	PADI1	PDCD1	PGR-AS1	PLA2G4C	POLL
NTSR1	PADI2	PDCD1LG2	PGRMC1	PLA2G5	POLQ
NUAK1	PADI4	PDCD2	PHACTR1	PLA2G6	POMC
NUCB2	PAEP	PDCD4	PHB	PLA2R1	POMP

NUDT1	PAFAH1B1	PDE2A	PHB2	PLAC1	PON1
NUDT2	PAG1	PDE5A	PHF2	PLAG1	POR
NUDT6	PAGR1	PDE8A	PHF20	PLAGL1	POSTN
NUF2	PAH	PDGFA	PHF20L1	PLAT	POT1
NUMA1	PAK1	PDGFB	PHGDH	PLAU	POTED
NUMB	PAK2	PDGFC	PHLDA1	PLAUR	POTEF
NUP214	PAK4	PDGFD	PHLDA3	PLB1	POU1F1
NUP62	PAK5	PDGFRA	PHLPP1	PLCB2	POU2F2
NUP88	PAK6	PDGFRB	PHLPP2	PLCD1	POU2F3
NUPR1	PALB2	PDIA2	PHRF1	PLCD4	POU4F2
NUS1	PALD1	**PDIA6**	PI3	PLCG1	POU5F1
NUSAP1	PALLD	PDIK1L	PIAS1	PLCG2	PPA1
NXT1	PAM	PDK1	PIAS3	PLD1	PPARA
OBP2A	PAN3	PDLIM1	PIBF1	PLD2	PPARD
OCA2	PANDAR	PDLIM4	PIEZO1	PLD3	PPARG
OCIAD1	PANX1	PDLIM5	PIF1	PLEK	PPARGC1A
OCLN	PAOX	PDLIM7	PIGK	PLEKHB1	PPARGC1B
ODAM	PAPOLG	PDPK1	PIGT	PLEKHG6	PPFIA1
ODC1	PAPPA	PDPN	PIGU	PLG	PPFIBP2
OGFOD1	PAQR3	PDS5B	PIH1D1	PLIN2	PPIA
OGG1	PARD3	PDXP	PIK3C2B	PLK1	PPIB
OGT	PARG	PDZK1	PIK3C3	PLK2	PPID
OLA1	PARK2	PDZK1IP1	PIK3CA	PLK3	PPIF
OLIG2	PARK7	PEA15	PIK3CB	PLK4	PPIG
OLR1	PARP1	PEAK1	PIK3CD	PLOD2	PPIP5K1
OPN1LW	PARP2	PEBP1	PIK3CG	PLP2	PPL
OPN5	PARP4	PEBP4	PIK3R1	PLPP1	PPM1D
PRKCDBP	PSMD4	PTPRO	RAD21	RBM14	RGS8
PRKCE	PSMD6	PTPRU	RAD23A	RBM14-RBM4	RGSL1
PRKCH	PSMD8	PTPRZ1	RAD23B	RBM3	RHAG
PRKCI	PSMD9	PTRH2	RAD50	RBM38	RHBDD2
PRKCQ	PSME1	PTTG1	RAD51	RBM39	RHBDF1

PRKCZ	PSME3	PTTG1IP	RAD51B	RBM4	RHBDF2
PRKD1	PSORS1C2	PTX3	RAD51C	RBM45	RHNO1
PRKD3	PTBP1	PUM1	RAD51D	RBM5	RHO
PRKDC	PTBP2	PVR	RAD52	RBMS3	RHOA
PRKG1	PTCH1	PVT1	RAD54B	RBMX	RHOB
PRL	PTEN	PWAR1	RAD54L	RBMY1A1	RHOBTB2
PRLR	PTENP1	PWAR4	RAD9A	RBMY1D	RHOC
PRM1	PTGDS	PXN	RAF1	RBMY2DP	RHOD
PRMT1	PTGER1	PYCARD	RAG2	RBP1	RHOQ
PRMT2	PTGER2	PYGM	RAI2	RBP2	RHOU
PRMT5	PTGER4	PYGO2	RALBP1	RBP4	RIBC2
PRMT6	PTGES	PYHIN1	RALGAPB	RBPJ	RICTOR
PRMT7	PTGES3	PYY	RALY	RBX1	RIEG2
PRNP	PTGFR	QPCT	RALYL	RCAN3	RIMS2
PROCR	PTGIR	QRSL1	RAN	RCC1	RIN1
PROM1	PTGIS	QSOX1	RANBP1	RCHY1	RIN2
PROS1	PTGS1	R3HDM2	RANBP9	RCN1	RINT1
PROX1	PTGS2	RA5	RAP1A	RECK	RIPK1
PRPF19	PTH	RAB11A	RAP1B	RECQL	RIPK2
PRPF31	PTH1R	RAB11FIP1	RAP1GDS1	RECQL4	RIPK3
PRPF38B	PTHLH	RAB11FIP3	RAP2A	RECQL5	RITA1
PRPF4B	PTK2	RAB1B	RAP2B	REG1A	RLN1
PRR5	PTK2B	RAB21	RAPGEF6	REG4	RLN2
PRRT1	PTK6	RAB22A	RAPH1	REL	RMDN1
PRRT2	PTK7	RAB25	RARA	RELA	RMDN2
PRRX1	PTMA	RAB27A	RARB	RELB	RMDN3
PRSS23	PTMAP4	RAB27B	RARG	RELN	RMND1
PRSS3	PTN	RAB2A	RARRES1	REM1	RMRP
PRSS3P2	PTOV1	RAB31	RARRES3	REN	RNASE3
PRSS50	PTP4A1	RAB35	RARS	REPS1	RNASEL
PRSS55	PTP4A2	RAB3A	RASA1	REPS2	RND1
PRSS8	PTP4A3	RAB3D	RASAL2	RERG	RND3

PRTN3	PTPA	RAB3GAP1	RASD1	RERGL	RNF11
PRUNE1	PTPN1	RAB40AL	RASEF	REST	RNF115
PSAP	PTPN11	RAB40B	RASGRF1	RET	RNF146
PSAT1	PTPN12	RAB40C	RASGRF2	RETN	RNF168
PSC	PTPN13	RAB5A	RASGRP3	REV1	RNF182
PSCA	PTPN14	RAB6A	RASSF1	REV3L	RNF19A
PSD4	PTPN2	RAB6B	RASSF10	REXO4	RNF2
PSEN1	PTPN22	RAB6C	RASSF2	RFC1	RNF31
PSEN2	PTPN23	RAB8A	RASSF3	RFC2	RNF41
PSG2	PTPN3	RABEP1	RASSF5	RFC3	RNF5
PSG5	PTPN6	RABEP2	RASSF7	RFC4	RNH1
PSIP1	PTPN9	RABGAP1L	RB1	RFWD2	RNPEP
PSMA1	PTPRA	RABGEF1	RB1CC1	RFX1	RNPS1
PSMA4	PTPRB	RABL6	RBBP4	RGCC	RNR1
PSMB10	PTPRC	RAC1	RBBP6	RGMB	RNR4
PSMB7	PTPRD	RAC2	RBBP7	RGN	RNU1-1
PSMB9	PTPRF	RAC3	RBBP8	RGS16	RNU1-4
PSMC3	PTPRG	RACGAP1	RBCK1	RGS17	RNU2-1
PSMC3IP	PTPRJ	RACK1	RBFOX2	RGS2	RNY1
PSMC4	PTPRK	RAD1	RBL1	RGS3	ROBO1
PSMC5	PTPRM	RAD17	RBL2	RGS4	ROCK1
PSMD10	PTPRN2	RAD18	RBM10	RGS6	ROCK2
RTKN	SCN1B	SETD2	SKCG-1	SLC34A2	SLX4
RTN1	SCN5A	SETD6	SKI	SLC35A2	SMAD1
RTN4	SCNN1A	SETD7	SKP2	SLC35A4	SMAD2
RTP4	SCP2	SETD8P1	SLC11A1	SLC35G1	SMAD3
RUNDC3B	SCPEP1	SF3A1	SLC12A2	SLC35G6	SMAD4
RUNX1	SCRIB	SF3B1	SLC12A3	SLC36A1	SMAD7
RUNX1T1	SCUBE2	SF3B3	SLC12A6	SLC37A1	SMARCA1
RUNX2	SDC1	SFN	SLC12A7	SLC38A1	SMARCA2
RUNX3	SDC2	SFR1	SLC12A9	SLC38A3	SMARCA4
RUVBL1	SDC3	SFRP1	SLC13A2	SLC38A6	SMARCA5

RXRA	SDC4	SFRP2	SLC13A5	SLC38A7	SMARCC1
RXRB	SDCBP	SFRP4	SLC16A1	SLC39A10	SMARCD3
RYR3	SDF2	SFRP5	SLC16A10	SLC39A2	SMARCE1
S100A1	SDF4	SFTPA1	SLC16A3	SLC39A6	SMC1A
S100A10	SDHA	SFTPA2	SLC16A4	SLC39A7	SMC4
**S100A11**	SDHB	SFXN1	SLC17A5	SLC39A9	SMC6
S100A12	SDHC	SGCG	SLC18A1	SLC3A2	SMG1
S100A14	SDHD	SGK1	SLC19A1	SLC40A1	SMN1
S100A16	SDPR	SGK3	SLC19A2	SLC44A1	SMN2
S100A2	SEC14L2	SGSM3	SLC19A3	SLC45A2	SMO
S100A4	SEC14L3	SH2B3	SLC1A5	SLC48A1	SMOX
S100A6	SEC16A	SH2D1A	SLC22A1	SLC4A11	SMPD1
S100A7	SEC23B	SH2D3A	SLC22A16	SLC4A7	SMPD2
S100A7A	SEL1L	SH2D3C	SLC22A17	SLC52A1	SMPD3
S100A8	SELE	SH3BP4	SLC22A18	SLC52A2	SMR3B
S100A9	SELENBP1	SH3GL1	SLC22A3	SLC5A5	SMUG1
S100B	SELENOF	SH3GL2	SLC22A5	SLC5A6	SMURF1
S1PR1	SELENOP	SH3GLB1	SLC23A2	SLC5A8	SMURF2
S1PR2	SELP	SH3KBP1	SLC25A10	SLC6A14	SMYD3
S1PR3	SEM1	SH3RF1	SLC25A16	SLC6A2	SMYD4
S1PR4	SEMA3A	SHARPIN	SLC25A20	SLC6A3	SMYD5
SACM1L	SEMA3C	SHBG	SLC25A21	SLC6A4	SNAI1
SAE1	SEMA3F	SHC1	SLC25A3	SLC6A5	SNAI2
SAFB	SEMA4C	SHC3	SLC25A37	SLC6A8	SNAPC1
SAFB2	SEMA4D	SHH	SLC25A41	SLC7A1	SNCA
SAGE1	SEMA6A	SHMT1	SLC25A43	SLC7A10	SNCG
SAI1	SEMA6B	SHOX2	SLC25A5	SLC7A11	SND1
SALL1	SEMG1	SIAH1	SLC25A52	SLC7A5	SNORD116@
SALL4	SENP1	SIAH1P1	SLC26A1	SLC9A1	SNORD14B
SARDH	SERPINA1	SIAH2	SLC26A2	SLC9A3R1	SNORD14C
SARS	SERPINA13P	SIGLEC7	SLC26A4	SLC9A3R2	SNORD14D
SARS2	SERPINA3	SIGMAR1	SLC26A8	SLCO1A2	SNORD14E

SART1	SERPINA4	SIK1	SLC27A2	SLCO1B1	SNORD15A
SART3	SERPINA5	SIL1	SLC27A4	SLCO1B3	SNORD28
SASH1	SERPINB1	SIM1	SLC28A1	SLCO2A1	SNORD35B
SAT1	SERPINB2	SIM2	SLC2A1	SLCO2B1	SNORD44
SAT2	SERPINB3	SIN3A	SLC2A10	SLCO3A1	SNORD50A
SATB1	SERPINB4	SIN3B	SLC2A12	SLCO4A1	SNRK
SCAF1	SERPINB5	SIPA1	SLC2A14	SLCO5A1	SNRPB
SCAF11	SERPINB6	SIRPA	SLC2A2	SLCO6A1	SNRPD1
SCAI	SERPINB9	SIRT1	SLC2A3	SLFN11	SNRPD3
SCARA3	SERPINE1	SIRT2	SLC2A4	SLIT1	SNRPN
SCARB1	SERPINE2	SIRT3	SLC2A4RG	SLIT2	SNTA1
SCD	SERPINF1	SIRT6	SLC2A5	SLIT3	SNW1
SCGB1A1	SERPINH1	SIRT7	SLC2A6	SLK	SNX9
SCGB1D2	SERTAD1	SIVA1	SLC2A9	SLN	SOAT1
SCGB2A1	SESN2	SIX1	SLC30A1	SLPI	SOCS1
SCGB2A2	SET	SIX2	SLC30A2	SLU7	SOCS2
SCGB3A1	SETBP1	SKA2	SLC33A1	SLURP1	SOCS3
SREBF1	STC1	TACC2	TENM4	TIMP1	TMPRSS13
SREBF2	STC2	TACC3	TEP1	TIMP2	TMPRSS3
SRF	STEAP1	TACR1	TERC	TIMP3	TMPRSS6
SRGAP2	STIM1	TACSTD2	TERF1	TIMP4	TMSB10
SRGAP3	STIM2	TADA3	TERF2	TINAGL1	TMSB15A
SRGN	STIP1	TAF5L	TERF2IP	TINF2	TMSB15B
SRI	STK11	TAF8	TERT	TIPARP	TMX2
SRM	STK17B	TAGLN2	TES	TIPIN	TNC
SRMS	STK25	TAL1	TESC	TIPRL	TNF
SRPK1	STK3	TAM	TET1	TJP1	TNFAIP1
SRPRB	STK39	TANC2	TET2	TJP3	TNFAIP2
SRRM2	STK4	TANK	TET3	TK1	TNFAIP3
SRSF1	STMN1	TAOK3	TEX101	TKT	TNFAIP8
SRSF2	STMN3	TAP2	TEX14	TKTL1	TNFRSF10A
SRSF3	STRAP	TARBP2	TF	TLE1	TNFRSF10B

SRSF5	STS	TARDBP	TFAM	TLE3	TNFRSF10C
SRSF6	STUB1	TAS2R13	TFAP2A	TLE4	TNFRSF10D
SRXN1	STX18	TAS2R38	TFAP2B	TLK1	TNFRSF11A
SRY	STX1A	TAS2R64P	TFAP2C	TLK2	TNFRSF11B
SSAV1	STXBP4	TAT	TFDP1	TLN1	TNFRSF12A
SSBP1	STYK1	TAZ	TFF1	TLR1	TNFRSF13C
SSR1	SUB1	TBC1D9	TFF2	TLR10	TNFRSF14
SSRP1	SUGP1	TBCC	TFF3	TLR2	TNFRSF18
SSSCA1	SULF1	TBCE	TFPI	TLR3	TNFRSF1A
SST	SULF2	TBK1	TFPI2	TLR4	TNFRSF1B
SSTR1	SULT1A1	TBL1X	TFR2	TLR5	TNFRSF25
SSTR2	SULT1A2	TBL1XR1	TFRC	TLR6	TNFRSF8
SSTR3	SULT1C2	TBL1Y	TGFA	TLR7	TNFRSF9
SSTR4	SULT1E1	TBP	TGFB1	TLR9	TNFSF10
SSTR5	SULT2A1	TBPL1	TGFB2	TM4SF1	TNFSF11
SSX2	SULT2B1	TBX1	TGFB3	TM6SF1	TNFSF12
SSX2B	SULT4A1	TBX2	TGFBI	TM7SF2	TNFSF12-TNFSF13
ST13	SUMO1	TBX3	TGFBR1	TMBIM6	TNFSF13
ST14	SUMO2	TBX4	TGFBR2	TMED10	TNFSF13B
ST18	SUMO3	TBXA2R	TGFBR3	TMED5	TNFSF14
ST2	SUPT20H	TBXAS1	TGM2	TMED7	TNFSF18
ST3GAL1	SUPV3L1	TCEA1	TGM3	TMED7-TICAM2	TNIK
ST6GALNAC1	SUSD2	TCEA2	TGM7	TMEFF1	TNIP1
ST6GALNAC2	SUSD3	TCEA3	THAP10	TMEFF2	TNK2
ST6GALNAC5	SUSD4	TCEAL1	THBS1	TMEM132D	TNKS
ST7	SUV39H1	TCF20	THBS2	TMEM132E	TNKS2
ST8SIA1	SUZ12	TCF21	THEG	TMEM135	TNMD
ST8SIA2	SVEP1	TCF3	THEMIS	TMEM14C	TNN
ST8SIA4	SYBU	TCF4	THEMIS2	TMEM158	TNNC1
STAG1	SYCE1L	TCF7	THOC1	TMEM173	TNNT1
STAP2	SYCP1	TCF7L2	THOP1	TMEM199	TNP1

STAR	SYK	TCHP	THRA	TMEM219	TNR
STARD10	SYN1	TCL1A	THRA1/BTR	TMEM25	TNS1
STARD13	SYNE1	TCL1B	THRB	TMEM33	TNS3
STARD3	SYNJ2	TCP1	THRSP	TMEM43	TNXB
STARD8	SYNJ2BP	TDGF1	THY1	TMEM45A	TOB1
STAT1	SYNM	TDGF1P3	TIAL1	TMEM54	TOMM40
STAT2	SYNPO	TDO2	TIAM1	TMEM70	TOP1
STAT3	SYT1	TEAD1	TICAM2	TMEM88	TOP2A
STAT4	TAAR5	TEC	TIE1	TMEM8B	TOP3A
STAT5A	TAB1	TEK	TIMELESS	TMOD1	TOPBP1
STAT5B	TAB2	TEKT4	TIMM17A	TMOD4	TOX
STAT6	TAC1	TELO2	TIMM50	TMPO	TOX3
STATH	TACC1	TENM3	TIMM8A	TMPRSS11D	TP53
TRPS1	UBE2B	USP6	WDR5	YME1L1	ZNF469
TRPV1	UBE2C	USP7	WDTC1	YPEL1	ZNF645
TRPV6	UBE2D3	USP9X	WEE1	YPEL2	ZNF652
TSACC	UBE2E2	USPL1	WIF1	YPEL3	ZNF654
TSHR	TYMS	UPK1B	VIP	WNT7B	ZACN
TSHZ1	TYR	UPP1	VIPR1	WNT9A	ZAR1L
TSHZ2	TYRP1	UPRT	VIPR2	WRAP53	ZBED1
TSHZ3	UBA2	UQCRFS1	VMP1	WRN	ZBP1
TSLP	UBASH3B	UQCRH	VNN2	WT1	ZBTB10
TSPAN1	UBC	USE1	VPS39	WT1-AS	ZBTB24
TSPAN13	UBE2E3	USF1	VPS4B	WWC1	ZBTB32
TSPO	UBE2I	USF2	VPS51	WWOX	ZBTB33
TSPY1	UBE2K	USP1	VRK1	WWP1	ZBTB4
TSPY10	UBE2L6	USP13	VRK2	WWTR1	ZBTB7A
TSPY3	UBE2S	USP17L2	VTA1	XBP1	ZC3H11A
TSPY4	UBE2T	USP2	VTCN1	XBP1P1	ZC3H12A
TSPYL5	UBE3A	USP28	VTN	XCL1	ZC3HAV1
TSTA3	UBL5	USP33	VWA5A	XDH	ZDHHC7
TSTD1	UBQLN1	USP4	VWF	XIAP	ZEB1

TTC39A	UBR5	USP44	WARS	XIST	ZEB2
TTC4	UCA1	UTP20	WARS2	XK	ZFAS1
TTK	UCHL1	UTRN	WAS	XKR3	ZFHX3
TTN	UCMA	UTS2	WASF1	XPA	ZFP36
TTR	UCN	UVRAG	WASF3	XPC	ZFP36L1
TUBA1B	UCP1	VAMP7	WASL	XPO1	ZFP36L2
TUBA4A	UCP2	VAMP8	WBP2	XPR1	ZFP69B
TUBB	UFM1	VANGL1	WDHD1	XRCC1	ZFP82
TUBB2B	UGCG	VANGL2	WDR20	XRCC2	ZFX
TUBB3	UGT1A	VAPB	WDR26	XRCC3	ZFYVE26
TUBB4B	UGT1A1	VASH1	WDR3	XRCC4	ZFYVE9
TUBD1	UGT1A10	VASP	WIPF1	XRCC5	ZHX2
TUBE1	UGT1A3	VAT1	WISP1	XRCC6	ZMIZ1
TUBG1	UGT1A6	VAV1	WISP2	XRCC6P5	ZMYND11
TUBG2	UGT1A8	VAV2	WISP3	YAP1	ZNF131
TUBGCP4	UGT1A9	VAV3	WLS	YARS	ZNF217
TUSC2	UGT2B11	VCAM1	WNK1	YBX1	ZNF224
TWF1	UGT2B15	VCAN	WNT1	YBX3	ZNF236
TWIST1	UGT2B17	**VCP**	WNT10B	YES1	ZNF24
TWIST2	UGT2B4	VDAC1	WNT11	YKT6	ZNF300
TWSG1	UGT2B7	VDR	WNT16	YLPM1	ZNF32
TXN	UGT8	VEGFA	WNT2	YPEL4	ZNF331
TXN2	UHRF1	VEGFB	WNT2B	YPEL5	ZNF35
TXNDC15	UHRF1BP1	VEGFC	WNT3	YWHAE	ZNF350
TXNDC5	UHRF2	VEGFD	WNT3A	YWHAG	ZNF365
TXNIP	UIMC1	VEZF1	WNT4	YWHAH	ZNF366
TXNRD1	ULK1	VGLL1	WNT5A	**YWHAQ**	ZNF395
TXNRD2	UMOD	VHL	WNT5B	**YWHAZ**	ZNF398
TYK2	UMPS	VHLL	WNT6	YY1	ZNF410
TYMP	UNC45A	**VIM**	WNT7A	YY1AP1	ZNF423
