# Supplementary material for: Characterization of the molecular changes associated with the overexpression of a novel epithelial cadherin splice variant mRNA in a breast cancer model using proteomics and bioinformatics approaches: identification of changes in cell metabolism and an increased expression of lactate dehydrogenase B
Source: Cancer Metab. 2019 May 9;7:5. doi: 10.1186/s40170-019-0196-9 (PMC6507066; doi:10.1186/s40170-019-0196-9)
Supplement: Supplementary file 6 — Table S1. Histological and molecular characteristics of breast tumor tissue samples. Description table in which the histological type and grade, the presence or absence of metastasis, the expression pattern of ER, PR and HER2, the percentage (%) of Ki-67 staining, as well as the molecular subtype of 21 breast tumor tissues, are detailed. (DOCX 17 kb) [file 40170_2019_196_MOESM6_ESM.docx]

**Additional Table 1: Histological and molecular characteristics of breast tumor tissue samples.**

| ***Sample*** | ***Histological Type*** | ***Histological Grade*** | ***Metastasis*** | ***ER*** | ***PR*** | ***HER2*** | ***Ki-67*** | ***Molecular Subtype*** |
| --- | --- | --- | --- | --- | --- | --- | --- | --- |
| T1 | Invasive lobular carcinoma | II | No | Positive | Positive | Positive (Score 3) | 10% | Luminal B HER2^+^ |
| T2 | Invasive ductal carcinoma, NOS | I | No | Positive | Positive | Negative (Score 0) | 10% | Luminal A |
| T3 | Invasive lobular carcinoma | II | No | Positive | Positive | Negative (Score 0) | 3% | Luminal A |
| T4 | Invasive ductal carcinoma, NST | II | Yes | Positive | Positive | Negative (Score 0) | 15% | Luminal B HER2^-^ |
| T5 | Invasive ductal carcinoma, NST | III | No | Positive | Positive | Negative (Score 0) | 16% | Luminal B HER2^-^ |
| T6 | Invasive ductal carcinoma, NST | II | No | Positive | Positive | Negative (Score 0) | 3% | Luminal A |
| T7 | Invasive ductal carcinoma, papilar | III | No | Positive | Positive | Negative (Score 0) | 5% | Luminal A |
| T8 | Invasive ductal carcinoma, NST | III | Yes | Positive | Positive | Negative (Score 0) | 16% | Luminal B HER2^-^ |
| T9 | Invasive ductal carcinoma, NOS | II | No | Positive | Positive | Negative (Score 0) | 8% | Luminal A |
| T10 | Invasive ductal carcinoma, NOS | III | Yes | Negative | Negative | Positive (Score 3) | 20% | HER2^+^ |
| T11 | Invasive ductal carcinoma, NST | III | No | Negative | Negative | Negative (Score 1) | 20% | Basal (Triple Negative) |
| T12 | Invasive carcinoma, NST | III | No | Positive | Positive | Negative (Score 1) | 15% | Luminal B HER2^-^ |
| T13 | Invasive ductal carcinoma, NST | I | No | Positive | Positive | Negative (Score 1) | 10% | Luminal A |
| T14 | Invasive ductal carcinoma, NST | III | Yes | Positive | Positive | Negative (Score 0) | 5% | Luminal A |
| T15 | Invasive ductal carcinoma, NOS | III | Yes | Negative | Negative | Positive (Score 3) | 25% | HER2^+^ |
| T16 | Invasive ductal carcinoma, NST | III | Yes | Positive | Positive | Negative (Score 1) | 40% | Luminal B HER2^-^ |
| T17 | Invasive lobular carcinoma | III | No | Positive | Positive | Negative (Score 0) | 40% | Luminal B HER2^-^ |
| T18 | Invasive ductal carcinoma, NST | II | No | Positive | Positive | Negative (Score 0) | 6% | Luminal A |
| T19 | Invasive ductal carcinoma, medular | III | No | Negative | Negative | Negative (Score 1) | 80% | Basal (Triple Negative) |
| T20 | Infiltrating ductal carcinoma | I | No | Positive | Positive | Negative (Score 0) | 18% | Luminal B HER2^-^ |
| T21 | Infiltrating ductal carcinoma | III | Yes | Positive | Negative | Negative (Score 1) | 60% | Luminal B HER2^-^ |

Description table in which the histological type and grade, the presence or absence of metastasis, the expression pattern of ER, PR and HER2, the percentage (%) of Ki-67 staining, as well as the molecular subtype of 21 breast tumor tissues, are detailed.
