# Supplementary material for: Characterization of the molecular changes associated with the overexpression of a novel epithelial cadherin splice variant mRNA in a breast cancer model using proteomics and bioinformatics approaches: identification of changes in cell metabolism and an increased expression of lactate dehydrogenase B
Source: Cancer Metab. 2019 May 9;7:5. doi: 10.1186/s40170-019-0196-9 (PMC6507066; doi:10.1186/s40170-019-0196-9)
Supplement: Supplementary file 7 — Table S2. Transcripts expression analysis of E-cadherin variant and LDHB in human breast tumor samples. Quantitative expression analysis of E-cadherin variant and LDHB by real time PCR in 21 human breast tumor tissues. Samples were numbered and ordered in an ascending form according with the 2-ΔCt values obtained for the E-cadherin variant mRNA. (DOCX 15 kb) [file 40170_2019_196_MOESM7_ESM.docx]

**Additional Table 2: Transcripts expression analysis of E-cadherin variant and LDHB in human breast tumor samples.**

| ***Sample*** | ***E-cadherin variant (2^-ΔCt^)*** | ***LDHB (2^-ΔCt^)*** |
| --- | --- | --- |
| T1 | 0.000078 | 0.062935 |
| T2 | 0.000097 | 0.128960 |
| T3 | 0.000106 | 0.097734 |
| T4 | 0.000126 | 0.011010 |
| T5 | 0.000148 | 0.068631 |
| T6 | 0.000163 | 0.059954 |
| T7 | 0.000219 | 0.042837 |
| T8 | 0.000279 | 0.046714 |
| T9 | 0.000281 | 0.018011 |
| T10 | 0.000288 | 0.134437 |
| T11 | 0.000353 | 0.376368 |
| T12 | 0.000445 | 0.143587 |
| T13 | 0.001072 | 0.116427 |
| T14 | 0.001076 | 0.013792 |
| T15 | 0.001190 | 0.384302 |
| T16 | 0.001432 | 0.249899 |
| T17 | 0.005355 | 0.060162 |
| T18 | 0.005839 | 0.133508 |
| T19 | 0.008461 | 0.107321 |
| T20 | 0.008490 | 0.123707 |
| T21 | 0.008669 | 0.290176 |
|  |  |  |

Quantitative expression analysis of E-cadherin variant and LDHB by real time PCR in 21 human breast tumor tissues. Samples were numbered and ordered in an ascending form according with the 2^-ΔCt^ values obtained for the E-cadherin variant mRNA.
